# Deficiency and excess of groundwater iodine and their health associations

**DOI:** 10.1038/s41467-022-35042-6

**Published:** 2022-11-29

**Authors:** Ruoqi Ma, Mingquan Yan, Peng Han, Ting Wang, Bin Li, Shungui Zhou, Tong Zheng, Yandi Hu, Alistair G. L. Borthwick, Chunmiao Zheng, Jinren Ni

**Affiliations:** 1 College of Environmental Sciences and Engineering, Peking University; Key Laboratory of Water and Sediment Sciences, Ministry of Education, Beijing, 100871 P. R. China; 2 Eco-environment and Resource Efficiency Research Laboratory, School of Environment and Energy, Peking University Shenzhen Graduate School, Shenzhen, 518055 P.R. China; 3 General Institute of Water Resources and Hydropower Planning and Design, Ministry of Water Resources, Beijing, 100120 P. R. China; 4 State Environmental Protection Key Laboratory of All Materials Fluxes in River Ecosystems, Peking University, Beijing, 100871 P. R. China; 5 Provincial Key Laboratory of Soil Environment Health and Regulation, Fujian Agriculture and Forestry University, Fuzhou, 350002 P. R. China; 6 Institute of Infrastructure and Environment, School of Engineering, The University of Edinburgh, Edinburgh, EH9 3JL UK; 7 School of Engineering, Mathematics and Computing, University of Plymouth, Plymouth, PL8 4AA UK; 8 State Environmental Protection Key Laboratory for Integrated Control of Groundwater and Surface Water Pollution in Watershed, Southern University of Science and Technology, Shenzhen, 518055 P. R. China

**Keywords:** Environmental impact, Environmental monitoring

## Abstract

More than two billion people worldwide have suffered thyroid disorders from either iodine deficiency or excess. By creating the national map of groundwater iodine throughout China, we reveal the spatial responses of diverse health risks to iodine in continental groundwater. Greater non-carcinogenic risks relevant to lower iodine more likely occur in the areas of higher altitude, while those associated with high groundwater iodine are concentrated in the areas suffered from transgressions enhanced by land over-use and intensive anthropogenic overexploitation. The potential roles of groundwater iodine species are also explored: iodide might be associated with subclinical hypothyroidism particularly in higher iodine regions, whereas iodate impacts on thyroid risks in presence of universal salt iodization exhibit high uncertainties in lower iodine regions. This implies that accurate iodine supply depending on spatial heterogeneity and dietary iodine structure optimization are highly needed to mitigate thyroid risks in iodine-deficient and -excess areas globally.

## Abstract (web summary)

Both iodine deficiency and excess could cause thyroid disorders. By creating a national map of groundwater iodine throughout China, the authors reveal the spatial responses of diverse health risks to iodine in continental groundwater.

## Introduction

Iodine is a micronutrient element essential in the production of thyroid hormones and important in energy metabolism, thermoregulation, and physical and mental development^1,2^. Insufficient or excessive ingestion of iodine can result in Iodine Deficiency Disorders (IDDs) or Iodine Excess Disorders (IEDs)^3^. From an epidemiological perspective, deficiency or excess of total iodine (TI) intake can lead to overt hyperthyroidism and hypothyroidism^3^. Iodine deficiency is often associated with nodular goiter^4^, whereas iodine excess is a common cause of subclinical hypothyroidism^3^. Although IDDs and their prevention remain of great concern^5^, increasing attention has recently been paid to IEDs because of emerging health problems from excessive iodine intake^1,6^.

Iodine in groundwater is unevenly distributed around the world^2^. Low iodine groundwater occurs mostly in the piedmont areas of China^7^. High iodine groundwater tends to be located in coastal plains^8^ and islands^9^, and is scattered elsewhere in paleo-ocean environments and certain inland basins in the Americas^10^, Europe^8^, Asia^7,9^, etc. Previous studies have focused on groundwater iodine in areas, mostly at smaller spatial scales^11^, where groundwater acts as the main source of drinking water^12^. The heterogeneous distribution of groundwater iodine results in diverse public health problems in different countries. For example, IDDs pose a global health problem that affects more than 30 countries around the world^13^. However, IEDs caused by high iodine groundwater have occurred in Denmark^14^, Chile^10^, Argentina^15^, Japan^9^, and China^7^. In the Anthropocene, spatial distribution of groundwater iodine has been subject to considerable change driven by natural variation and human interference.

Iodine speciation is of great significance to the biogeochemical cycle and health risk. In the hydro-biogeochemical cycle, iodine predominantly appears in oxidation state and reduction state, i.e. iodate (IO_3_^−^) and organo-iodine as well as iodide (I^−^)^2^. Reduction iodine (I^−^), with high mobility and bioavailability, is the major species under anoxic and reducing groundwater environments, especially in groundwater with high-iodine content^16^. I^−^ can be oxidized by disinfectants to generate iodinated disinfection byproducts (I-DBPs)^17^, which have much higher toxicity than their brominated and chlorinated analogues^18,19^. Oxidation iodine (IO_3_^−^ and organo-iodine), which often appear in a non-steady state, are more likely to exist under weak oxidizing thermodynamic conditions^16^ commonly found in low-iodine groundwater. IO_3_^−^ is more likely to bond with sediment rather than enrich groundwater^20^, and thus groundwater IO_3_^−^ rarely exceeds the threshold at which oxidative damage occurs^21^. Different organo-iodine compounds can also comprise a significant fraction of TI^16^. With regard to health risk assessment related to organo-iodine, total organo-iodine content has been proposed as a toxic substitute for I-DBPs in drinking water^19^, but the specific bioavailability and toxicity of natural organically bound iodine are presently unknown due to the complexity of its components and properties.

**Fig. 1:**
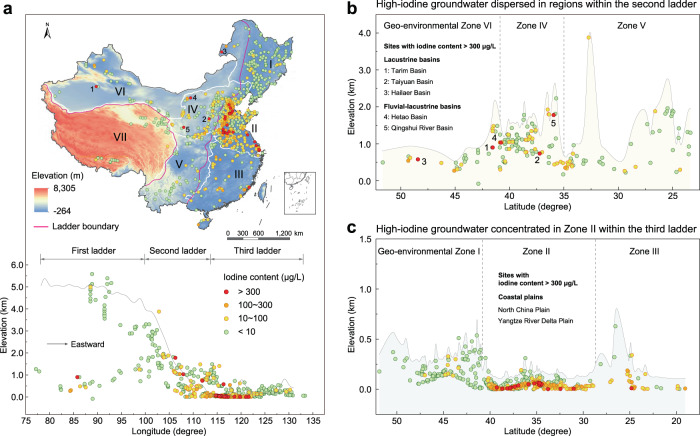
Spatial distribution of groundwater iodine in seven geo-environmental zones throughout China. **a** Spatial distribution of groundwater iodine, and its variation with elevation above mean sea level. Roman numerals I to VII indicate the different geo-environmental zones, namely: Northeast Plain-Mountain Zone (I), Huanghuaihai-Yangtze River Delta Plain Zone (II), South China Bedrock Foothill Zone (III), Northwest Loess Plateau Zone (IV), Southwest China Karst Rock Mountain Zone (V), Northwest Arid Desert Zone (VI), and Qinghai-Tibet Plateau Alpine Frozen Soil Zone (VII). Iodine content is classified into <10, 10 ~ 100, 100 ~ 300 and >300 μg/L levels, denoted by the colored symbols listed in the legend. In the lower panel, the gray line (the topographic profile of 35°N in North China) shows the variation in elevation with longitudinal distance eastward from the coast. **b**, **c** Characteristics of groundwater iodine profiles with latitudinal distance in the second (**b**) and third ladders (**c**) (captured using ArcGIS).

Here we provide the national map of groundwater iodine throughout China based on our first-hand monitored results (Fig. 1 and Supplementary Fig. 1). The uneven distribution of iodine and its species in groundwater not only mirrors long-term natural and anthropogenic effects, but also highlights the importance of iodine species in evoking potential thyroid risks in iodine-deficient and iodine-excess regions worldwide.

## Uneven spatial distribution of total iodine

We derive a groundwater iodine map across China (Fig. 1 and Supplementary Fig. 1), which exhibits large spatial variation in TI content (range 0.28 ~ 1006.25 μg/L, mean 47.08 μg/L, and median 7.29 μg/L). Current National Standards in China for water-borne iodine-deficient/excess endemic areas divide iodine content in groundwater into the following four categories: low, TI < 10 μg/L; medium, 10≥ TI > 100 μg/L; high, 100≥ TI > 300 μg/L; and very high, TI > 300 μg/L. Our sampling campaign (Supplementary Tables 1, 2) reveals that low iodine groundwater is extensively distributed throughout China (56.85% of the total), which is primarily attributed to insufficient iodine supply from magmatic and metamorphic rocks (which comprise >90% of the rock volume of the earth’s crust)^22^. High and very high iodine groundwater is mainly distributed (12.97% of the total) in the Huanghuaihai-Yangtze River Delta Plain Zone (II), South China Bedrock Foothill Zone (III), and Northwest Loess Plateau Zone (IV), among the seven geo-environmental zones in China (Fig. 1a). Previous studies also reported regionally high iodine groundwater in the foregoing zones (e.g., Hebei^13^, Guangdong^23^, and Shanxi^11^ provinces), consistent with the present findings.

The national distribution of groundwater iodine is profiled in terms of China’s three-ladder terrain^24^, characterized with descending elevation eastward from the first (>4000 m) at Qinghai-Tibet plateau, through the second (1000 ~ 2000 m) at northeast, southwest, and northwest China, to the third (<500 m) at eastern China (Fig. 1a). Interestingly, groundwater iodine content exhibits significantly negative correlation with elevation (*P* < 0.001) (Supplementary Fig. 2a, b), in accordance with previous hypotheses that elevation plays a role in influencing atmospheric exchange between land and ocean^22,25^.

At the first ladder, the Qinghai-Tibet Plateau acts as a typical iodine-deficient region with groundwater iodine content in the range 0.69 ~ 39.56 μg/L (mean 4.54 μg/L, median 3.40 μg/L). At the highest altitude, effects of the iodine-enriched marine atmosphere are restricted as the landscape changes from periglacial to fluvial environments^26^. Deeply buried sub-permafrost water in Qinghai-Tibet Plateau undergoes relatively short cycles due to high elevation, rugged terrain, steep groundwater gradient, and large flow rate^26^, all detrimental to iodine enrichment.

At the second ladder, groundwater iodine content is in the range 0.39 ~ 1006.25 μg/L (mean 28.09 μg/L, median 5.71 μg/L), where the geomorphology is dominated by both plateau/mountain and sedimentary basins. Based on the Local Moran’s Index (see Methods), 80% high-value outliers located at this ladder (Supplementary Fig. 2c) reflecting the characteristic of dispersed distribution, and dozens of high-iodine groundwater samples dispersed in inland regions, such as the Tarim, Taiyuan, and Hetao basins (Fig. 1b). Organic matter-rich lacustrine or fluvial sediments are mainly distributed in inland basins^11^, plains with river networks^27^, and paleochannels^28^. Such sediments provide favorable material conditions for iodine enrichment in sedimentary facies^29^. For example, the iodine content of sediment in Datong basin (0.18 ~ 1.46 μg/g)^30^ is comparable to that of marine sediment (0.03 ~ 2.54 μg/g)^29^, implying the iodine enrichment mechanism is similar in sedimentary basins and oceans. Paleochannel swing enhances the reducing environment, cation exchange, and low infiltration recharge of surface water, which can promote iodine release from iodine-rich aquifer sediment into groundwater^28^. Relatively sparse rainfall and intense evaporation at this ladder are also favorable to elements enrichment^31^.

**Fig. 2:**
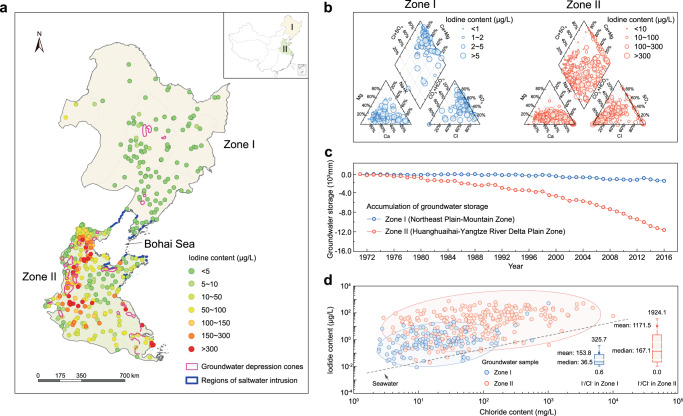
Geogenic and anthropogenic influences on groundwater iodine distribution in China. **a** Spatial distributions of groundwater iodine content in Zone I (Northeast Plain-Mountain Zone) and Zone II (Huanghuaihai-Yangtze River Delta Plain Zone). **b** Piper diagrams representing Na^+^-SO_4_^2−^ hydro-chemical type (blue circle) and Na^+^-Cl^−^ hydro-chemical type (red circle) of groundwater in Zones I and II. **c** Annual variability of groundwater storage accumulation (1971 ~ 2016) in Zones I and II. **d** Groundwater iodine enrichment factor (iodide content in μg/L versus chloride content in mg/L) in Zones I (*n* = 113) and II (*n* = 274). The box is bounded by the first and the third quartile with a horizontal line at the median and a hollow square at the mean, and whiskers extend to the maximum and minimum value in 1.5 times interquartile range.

The third ladder is the lowest morphogenetic region of China. It is the most centralized regarding high groundwater iodine distribution (range 0.28 ~ 982.30 μg/L) (Supplementary Fig. 2b, c) owing to its geographical proximity to the ocean. Notably, uneven distribution of groundwater iodine within this ladder (Fig. 1c) is exhibited by significant difference in iodine contents in the Northeast Plain-Mountain Zone (I) (median 1.51 μg/L) and Zone II (median 31.50 μg/L)^2,32,33^. In addition to the direct influence of marine atmosphere, sediment deposits during the periods of marine transgressions are considered as the main source of groundwater iodine, providing evidence for the existence of high iodine content in regions where organic matter-rich marine sediments are mainly distributed^34^ (Fig. 2a). Distinguished from Na^+^-SO_4_^2−^ type groundwater in Zone I related to the weathering of parent rocks, Na^+^-Cl^−^ is the dominant hydro-chemical type of high iodine groundwater in Zone II (Fig. 2b and Supplementary Table 3) which indicates significant characteristics of marine^13^. From geological and hydrogeological perspectives, the most stable species of inorganic iodine in seawater and groundwater are iodate and iodide, respectively. Due to its greater adsorption capability than iodide, iodate can accumulate in sediments during marine transgression. In the Quaternary Period, the fluctuation of sea level triggered several marine transgression events in coastal areas of the North China Plain (NCP) located in Zone II, which caused the accumulation of iodine-enriched sediments^13^. With marine transgression and seawater intrusion, iodine in sediment leached into groundwater, producing iodine-rich Na^+^-Cl^−^ type water in coastal areas^34^.

Natural geochemical processes cannot solely explain the huge difference in groundwater iodine between Zones I and II. The significant reduction in groundwater storage accumulation^35^ (see Methods) between Zone II (−11389.4 mm) and Zone I (−1358.0 mm) (Fig. 2c and Supplementary Table 4) reflects severe overexploitation of groundwater during 1971 ~ 2016 in Zone II (Supplementary Fig. 3, and Supplementary Tables 5–7). The enhanced compaction of aquitards and land subsidence associated with a decline in groundwater level promote the release of iodine-rich pore water from sediment into groundwater, resulting in high iodine groundwater in Zone II^13^. The iodate in sediments can be changed to iodide under the reducing conditions in deep confined aquifers, and iodide may be then desorbed from soils to pore water in sediments, causing elevated concentration of iodide in pore water. Compared with iodine, chloride can hardly be retained in the solid phase during marine transgression, resulting in the retention of iodide over chloride in sediments. Using the iodine enrichment ratio, defined as iodide/chloride (I^−^/Cl^−^)^13^, to indicate the origin of iodine in groundwater (Fig. 2d), the much larger value of I^−^/Cl^−^ in Zone II (mean 1171) than that in seawater (e.g., 2 in the Bohai sea)^13^ suggests a high likelihood of iodine release from iodine-rich transgressive sediments to groundwater^13^. Compared with that in Zone I (I^−^/Cl^−^ range 0.6 ~ 325.7), I^−^/Cl^−^ in Zone II (0.0 ~ 1924.1, Fig. 2d) is closer to the pore water (3 ~ 1020)^13^. Evidence for anthropogenic enhancement of pore water release to groundwater is provided by the much higher iodine content in phreatic groundwater post-2000 (124.50 ~ 481.21 μg/L) near the coastal area of Zone II compared with that prior to 1970 (27.20 μg/L)^36^. Since 1980, the accumulated iodine contribution of compacted pore water has become significant (219 ~ 572 μg/L)^13^ due to the acceleration of groundwater exploitation in the NCP.

## Spatial distribution of iodine speciation

Enrichment of iodine and its species transformation in groundwater are affected by deposition conditions, geochemical conditions (e.g., oxidation reduction potential and pH), and human activities^25,34^ (Supplementary Fig. 4), suggesting spatial differentiation of iodine species might exist in varying geo-environmental zones (Supplementary Figs. 5, 6).

Iodide (I^−^, 0.01 ~ 956.70 μg/L, mean 37.46 μg/L, and median 2.12 μg/L) is the dominant species (48.94% ± 31.43% of TI) in the reduction environment^37^. I^−^ is strongly correlated with TI (R^2^ = 0.97, *P* < 0.001) (Supplementary Fig. 6a, b) in groundwater, consisting with greater proportion of I^−^ (86.96% ± 12.49% of TI) occurred in iodine-excess groundwater (TI > 300 μg/L) under reducing environments.

Oxidation iodine species (IO_3_^−^ and organo-iodine) more occur in oxidizing and organic rich environments^2,17^. The stronger absorption capacity of oxidation iodine onto mineral surfaces^20^ than I^−^ makes the species to be dominant (contributing 64.30% ± 26.40% to total iodine content) in iodine-deficient areas (TI < 10 μg/L). The dispersed distribution of high iodate groundwater under varying geo-environmental zones is reflected by a poor correlation between IO_3_^−^ and TI (R^2^ = 0.19, *P* < 0.001) (Supplementary Fig. 5c and Supplementary Fig. 6b). Groundwater IO_3_^−^ (mean 4.46 μg/L, median 1.64 μg/L, and 28.36% ± 24.80% of TI) is therefore mainly distributed in sedimentary basins and ancient channels with organic matter-rich sediment within Zone IV (median 2.41 μg/L) and the Northwest Arid Desert Zone (VI) (median 2.12 μg/L) (Supplementary Fig. 6a). Irrigation and groundwater exploitation may also modify the redox potential and contribute to the existence of IO_3_^−^ especially in phreatic water^13^. Organo-iodine with a relatively lower content (mean 4.97 μg/L and median 1.23 μg/L) contributes the minimum proportion of TI (22.70% ± 20.63%) in groundwater, mainly distributed in weakly oxidizing and organic rich environments. In general, high groundwater organo-iodine primarily occurs in the NCP (mean 8.88 μg/L and median 2.40 μg/L) (Supplementary Fig. 6a). Greater groundwater organo-iodine levels in the Yangtze River Delta Plain (median 7.18 μg/L) and Lianghu Plain (median 8.45 μg/L) (Supplementary Fig. 5d) also reflects the consequences from active interactions between surface water and groundwater due to human impacts^2,23,38^.

A vertical view on groundwater iodine in terms of groundwater-depth is helpful to understand the higher proportion (mean 59.39%, median 70.08%) of oxidation iodine that presents a monotonically decreasing trend with depth of reconstructed wells (particularly phreatic water, see Supplementary Fig. 6c and Supplementary Table 8). With increase in well depth, the oxidation iodine content of groundwater in newly constructed wells firstly increases due to rainfall, irrigation, and microbial oxidation processes^38^, and then decreases in a strong reducing environment^11^. For confined water in newly constructed wells (Supplementary Fig. 6c), the highest correlation between oxidation iodine content and well depth (R^2^ = 0.93, *P* < 0.001) is observed.

## Health effects of iodine in groundwater

Severe deficiency or excess of iodine can induce failure of thyroid regulating mechanisms^39^. Prevalence of overt hypothyroidism or hyperthyroidism follows a U-shaped curve with increase in iodine nutrient level evaluated as urine iodine content (UIC)^39^ (Supplementary Fig. 7a). As a major drinking water source, groundwater could be the principal source of dietary iodine for some populations in regions where drinking water iodine intake (DI_W_) (see Methods) is higher than dietary iodine intake through universal salt iodization (USI) (Supplementary Fig. 8), and has been associated with various thyroid diseases^39^. Assuming groundwater iodine as sole source of drinking water iodine intake which contributes to ~20% of dietary iodine intake^40^, non-carcinogenic risk is evaluated in terms of iodine deficiency (R_D_) or excess (R_E_) based on hazard quotient (HQ) (see Methods) via exposure route of drinking water (Supplementary Fig. 9). The value of R_D_ (mean 2.62) is greater than 1 for 52% of monitored wells, suggesting a wide distribution of risk related to iodine deficiency (Supplementary Fig. 9a, b). Risk of iodine excess appears in only 35% monitored wells, mostly concentrated in Zone II (mean R_E_ 5.74) and Zone III (mean R_E_ 3.02) (Supplementary Fig. 9c, d, and Supplementary Table 9). In addition, high iodine groundwater scattered in the Northwest Loess Plateau Zone (IV) (mean R_E_ 1.49) and the Northwest Arid Desert Zone (VI) (mean R_E_ 2.88) is largely attributed to lacustrine sediment.

**Fig. 3:**
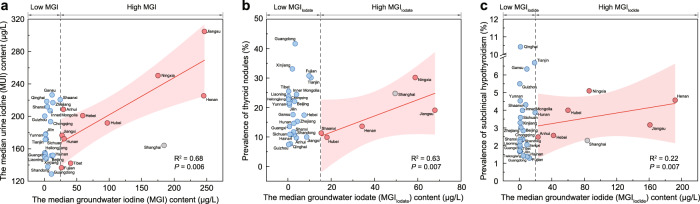
Correlations of thyroid diseases with groundwater iodine and its species in 36 cities from 31 provinces of China. **a** Correlation between the median urine iodine (MUI) and median groundwater iodine (MGI) contents (two-sided *t*-test). **b** Correlation between the prevalence of thyroid nodules and median groundwater iodate (MGI_Iodate_) (two-sided *t*-test). **c** Correlation between the prevalence of subclinical hypothyroidism and median groundwater iodide (MGI_Iodide_) (two-sided *t*-test). The pink lines (or shades) show the regression results (or the 95% prediction intervals) of the concerned variables. The black dotted lines demarcate the domains of lower and higher MGI (critical value 25 μg/L), MGI_Iodate_ (critical value 15 μg/L) and MGI_Iodide_ (critical value 20 μg/L). Gray circles represent Shanghai without utilizing groundwater.

Based on the relation between median urine iodine (MUI) and median groundwater iodine level (MGI) from 36 sampled cities in 31 provinces of China (Fig. 3a and Supplementary Table 10), we further test the roles of groundwater iodine with respect to the population iodine nutritional level (median urine iodine, MUI). In the relatively low-iodine regions (MGI < 25 μg/L, blue circles in Fig. 3a), MUI scattered greatly showing no correlation with MGI, suggesting the level of iodine nutrition might depend more on salt iodine and dietary iodine levels than on groundwater iodine under the background of the USI. In the relatively high-iodine groundwater regions (“hotspot” with MGI > 25 μg/L, red circles in Fig. 3a) with larger proportion of groundwater utilization, greater correlation between MUI and MGI (R^2^ = 0.68, *P* = 0.006) is found, indicating higher dependence of iodine nutritional levels on groundwater iodine levels. Similarly, we describe the changes in prevalence of thyroid diseases potentially associated with iodine species (Fig. 3b, c) in groundwater. Although toxicological analyses suggested^4,41^ that iodine species could disrupt the normal physiological process of the thyroid gland in different ways causing specific diseases, more studies are still needed to provide convincing interpretations.

**Fig. 4:**
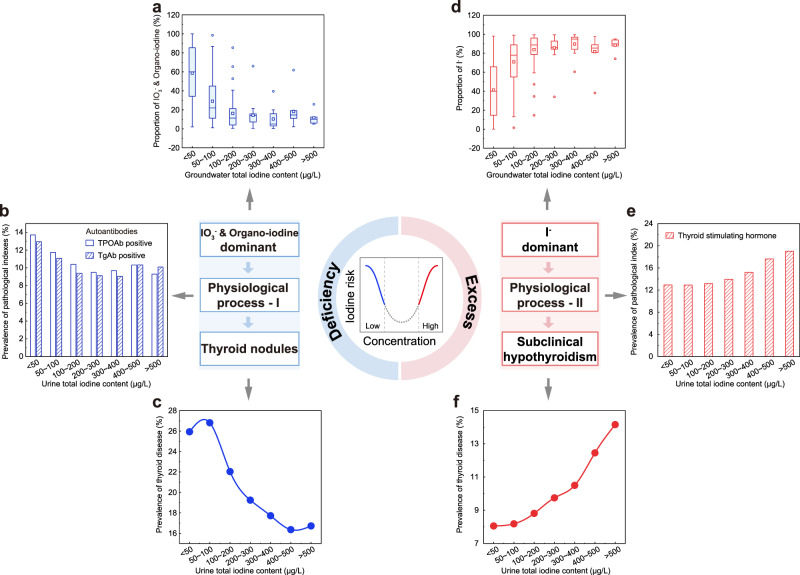
Potential risks posed by iodine species to human health. **a** Box plot showing proportions of IO_3_^−^ and organo-iodine in groundwater with different levels of total iodine content (*n* = 686). The box is bounded by the first and the third quartile with a horizontal line at the median and a hollow square at the mean, and whiskers extend to the maximum and minimum value in 1.5 times interquartile range. **b** Histogram depicting prevalence of thyroid autoantibodies (TPOAb and TgAb) with different levels of urine total iodine content. **c** Prevalence of thyroid nodules with different levels of urine total iodine content. **d** Box plot showing proportions of I^−^ in groundwater with different levels of total iodine content (*n* = 686). The box is bounded by the first and the third quartile with a horizontal line at the median and a hollow square at the mean, and whiskers extend to the maximum and minimum value in 1.5 times interquartile range. **e** Prevalence of thyroid stimulating hormone (TSH) with different levels of urine total iodine content. **f** Prevalence of non-autoimmune subclinical hypothyroidism with different levels of urine total iodine content.

Thyroid nodules (TNs) appear more prevalent in iodine-deficient regions where high proportion of IO_3_^−^ frequently occurs in groundwater (Fig. 4a–c). IO_3_^−^ is the main form of iodine in the USI, and the added IO_3_^−^ is mostly converted into I^−^ and I_2_ during cooking due to the presence of reductants in the food^42^. Since boiling could not remove the IO_3_^−^ in groundwater^42^, another potential source of ingesting IO_3_^−^ would be from drinking groundwater. To understand the possible effects of groundwater IO_3_^−^ intake on thyroid diseases, correlation analysis is performed on TNs prevalence and median groundwater IO_3_^−^ (MGI_Iodate_). In the relatively lower IO_3_^−^ regions (MGI_Iodate_<15 μg/L, blue circles in Fig. 3b), the scattered distribution of the prevalence of TNs suggests that the TNs might depend more on salt iodine and dietary iodine levels than on groundwater iodate because of the USI. In the regions with relatively higher IO_3_^−^ groundwater (MGI_Iodate_>15 μg/L, red circles in Fig. 3b) and greater proportion of groundwater utilization^43^, closer correlation between TNs and MGI_Iodate_ (R^2^ = 0.63, *P* = 0.007) suggests potential association of groundwater IO_3_^−^ with high prevalence of TNs. For example, the prevalence of TNs in Xuzhou (19.06%) (the sampled city in Jiangsu province) is higher than that in Xinxiang and Kaifeng (13.61%) (sampled cities in Henan province), in spite of their similar iodized salt coverage (98.26 ~ 99.46%) and salt iodine level (25 ~ 30 mg/kg), possibly related to higher groundwater IO_3_^−^ in Xuzhou (67.94 μg/L) than Henan (34.16 μg/L).

To understand the relevance of TNs to IO_3_^−^ exposure, investigations from the toxicological and epidemiological perspectives are often very useful. For example, experiments on thyroid tumor cells in vitro revealed that IO_3_^−^ at a dose of 127 μg/L could effectively enhance the proliferation of human thyroid carcinoma cells, which could be a risk factor for thyroid cancer, a specific form of TNs^41^. Moreover, animal studies indicated that chronic exposure to high water iodine from IO_3_^−^ could induce hypothyroidism with significant morphology changes in female Wistar rats^44^. Regional and national epidemiological studies^39,45^ also reported the correlation between higher cumulative incidence of nodular goiter or Graves’ disease and lower population iodine nutritional level (Fig. 4c and Supplementary Fig. 7b). Health risks related to organo-iodine are difficult to assess due to their complexity. Previous studies have indicated that xenobiotic organo-iodine (like amiodarone and erythrosine) is a risk factor for goiter^46^, whereas biogenic organo-iodine (e.g., tyrosine derivative 3,5-diiodotyrosine) has displayed protective effects on thyroid cells in animal experiments^47^.

Subclinical hypothyroidism occurs more frequently in iodine-excess regions where I^−^ is the major iodine species in groundwater (Fig. 4d–f). Animal studies^4^ provided an explicit pathological mechanism, whereby chronic high I^−^ intake could inhibit type 2 deiodinase activity in the pituitary, causing increase in serum thyroid stimulating hormone (Fig. 4e) reflected by high occurrence frequency of subclinical hypothyroidism (Fig. 4f), confirmed by epidemiological data^39,48^. Unlike autoimmune diseases caused by iodine deficiency, subclinical hypothyroidism induced by high I^−^ intake only presents itself in non-autoimmune individuals^39^ (Supplementary Fig. 7c). In the high iodine regions with larger proportion of groundwater utilization, the correlation between median groundwater I^−^ (MGI_Iodide_) and prevalence of subclinical hypothyroidism (R^2^ = 0.22, *P* = 0.007) (Fig. 3c) suggests the impact of high groundwater-iodide (MGI_Iodide_>20 μg/L, red circles in Fig. 3c) on IEDs.

Considering the implementation of USI policy in China, we point out the contribution of groundwater iodine intake to population iodine nutrition level at hotspots. Since the true relation between exposure and disease might be distorted by the inability to control for confounding variables^49^, more influencing factors should be considered to identify the potential correlation between iodine speciation and the prevalence of thyroid diseases based on well-designed epidemiological and eco-environmental studies.

## Recommendations for iodine-induced risk management in groundwater

**Fig. 5:**
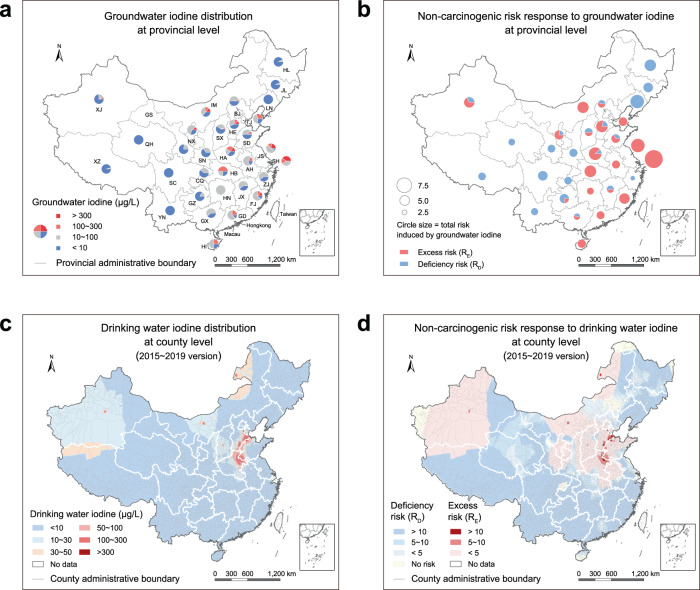
Spatial response of non-carcinogenic risk to groundwater iodine at provincial, and county level. **a** Groundwater iodine distribution at provincial level in China. Capital letters indicate different provinces, namely: Anhui (AH), Beijing (BJ), Chongqing (CQ), Fujian (FJ), Guangdong (GD), Gansu (GS), Guangxi (GX), Guizhou (GZ), Henan (HA), Hubei (HB), Hebei (HE), Hainan (HI), Heilongjiang (HL), Hunan (HN), Inner Mongolia (IM), Jilin (JL), Jiangsu (JS), Jiangxi (JX), Liaoning (LN), Ningxia (NX), Qinghai (QH), Sichuan (SC), Shandong (SD), Shanghai (SH), Shaanxi (SN), Shanxi (SX), Tianjin (TJ), Xinjiang (XJ), Tibet (XZ), Yunnan (YN), Zhejiang (ZJ). **b** Non-carcinogenic risks response to groundwater iodine at provincial level in China. **c** Drinking water iodine distribution at county level in China (2015 ~2019 water supply scheme). **d** Non-carcinogenic risks response to drinking water iodine at county level in China (2015 ~2019 water supply scheme).

It has been reported that globally about 2 billion people suffer endemic goiter solely from iodine deficiency^50^. Since the 1940s, salt iodization has been introduced as a sustainable strategy to improve the population level iodine intake around the world. Although the USI policy recommended by the World Health Organization (WHO) has led to significant progress in preventing IDDs, there remain concerns due to the large regional disparities in iodine status hidden by the national survey. For example, China has been identified as a country with adequate iodine nutrition by the Iodine Global Network (IGN), while thyroid disease induced by environmental iodine differences still affects about 0.5 billion adults^39^. In other words, the USI policy did help to reduce the large number of people who suffered deficient iodine intake, but at the same time puzzled with new problems arisen from excessive iodine intake. For a balanced iodine provision under varying dietary frameworks, the optimal scheme should be designed based on the status of iodine and its species in iodine-deficient or -excess areas (Fig. 5a).

Differential iodine provision in varying regions is strongly recommended given the diverse thyroid risks relevant to spatially heterogeneous distribution of groundwater iodine. For example, at provincial level, the risk from groundwater iodine deficiency is more likely to occur in Liaoning, Jilin, Heilongjiang, Xinjiang, Qinghai, Tibet, Gansu, Shaanxi, Sichuan, Guizhou, and Yunnan, whereas iodine excess risk is largely concentrated in Tianjin, Hebei, Shandong, Henan, Anhui, Hunan, Hubei, Jiangsu, Shanghai, Fujian, and Guangdong (Fig. 5b, and Supplementary Tables 11, 12). In regions of severe iodine deficiency (e.g., Sichuan, Yunnan, Liaoning, and Xinjiang in China), the coverage of adequately iodized salt (iodization level: 25 ~ 30 mg/kg) is required to be maintained above 90%^51^. To achieve the global target of sustainable elimination of IDDs, the USI policy of all food-grade salt is essential in these regions in addition to advanced monitoring and effective evaluation of iodine nutrient levels^51^. For sensitive populations in localized areas, other complementary, proven strategies (such as the distribution of iodized oil capsules) are also needed to maintain the required iodine nutrition level, particularly for school-age children (UIC: 100 ~ 199 μg/L) and pregnant/lactating women (UIC: 150 ~ 249 μg/L)^52^. In iodine-excess regions (e.g., Shanghai, Jiangsu, Hunan, Hubei, Anhui, Tianjin Fujian, and Henan), residents with an over-intake of iodine cannot be relieved by the supply of non-iodized salt alone^51^. Under such circumstances, water quality must be improved through advanced deiodination technologies such as adsorption^53^, membrane separation^54^, and capacitive deionization^55^. Otherwise, alternative water sources would be needed via surface or foreign water exploitation^56^. Based on the national water supply scheme (2015 ~ 2019), we identify the percentage of groundwater utilization in drinking water and generate a national map for drinking water iodine at county level (Fig. 5c and Supplementary Table 13). These results agree satisfactorily with the actual drinking water iodine distribution in a most recently survey^57^ (Supplementary Fig. 10 and Supplementary Table 14), indicating the significant contribution of groundwater iodine to drinking water iodine levels because of much lower iodine concentration in surface water. In some regions (e.g., Shanghai, Jiangsu, Hunan, and Hubei) with iodine-excess groundwater, the natural abundance of surface water has reduced dependence on groundwater and thus the iodine-induced risk (Fig. 5d). In rural areas of these regions, implementation of the USI policy is still necessary, together with surface water purification implemented by means of centralized water supply systems.

Species differential iodine provision concerns thyroid risks potentially associated with iodine speciation. Noting the reported relationship between occurrence of autoimmune thyroid disease and high content of IO_3_^−^ and/or organo-iodine^39,58^, with 96.4% of added KIO_3_ converted to I^−^ and I_2_^42^, iodized salt would also help reduce the relative contributions of these species to TI^41,58^ in sensitive areas mainly distributed in Yunnan, Xinjiang, Qinghai, Gansu, and Tibet (Supplementary Fig. 11a–c, and Supplementary Table 15). Moreover, household treatments (e.g., boiling and filtration) could selectively remove organo-iodine and/or IO_3_^−^ from drinking water^59^. In iodine-excess regions, deiodination and/or the exploitation of alternative water sources would also be beneficial for reducing the incidence of subclinical hypothyroidism due to high I^−^ intake, notably in Jiangsu, Anhui, and Henan (Supplementary Fig. 11d–f, and Supplementary Table 15).

**Fig. 6:**
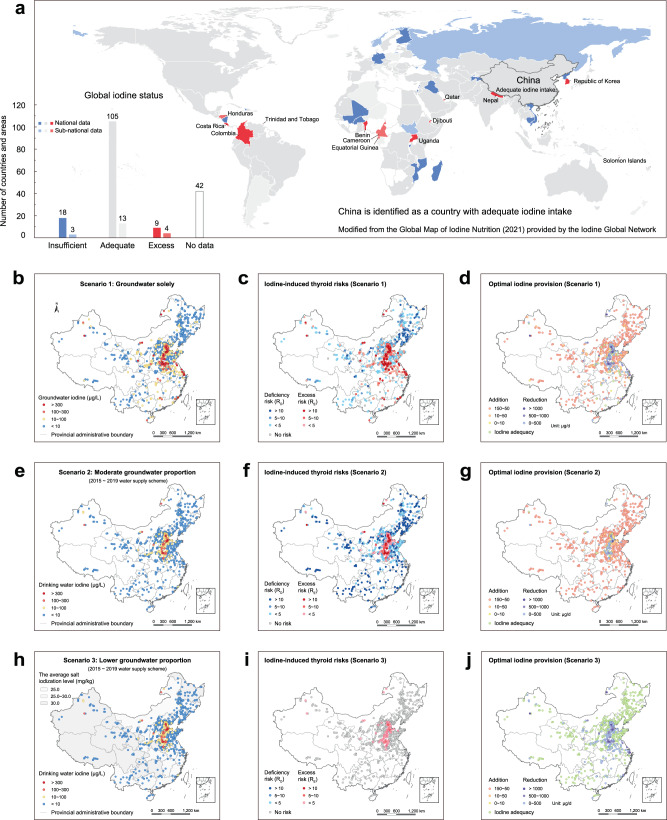
Optimal iodine provision with consideration of groundwater and iodized salt contribution in the total dietary iodine intake. **a** Iodine nutrition map for 194 WHO Member States (2021) according to the Iodine Global Network (https://www.ign.org/scorecard.htm). National median urine iodine content in school-age children <100 µg/L, 100 ~ 299 µg/L, and ≥300 µg/L represent insufficient, adequate, and excess iodine intake in the general population. **b** Scenario 1 represents groundwater iodine as the sole source of drinking water iodine nationwide. **c** Iodine-induced thyroid risks under Scenario 1. **d** Optimal iodine provision for reducing thyroid risks under Scenario 1. **e** Scenario 2 represents groundwater as partial source in drinking water utilization according to the water supply scheme (2015 ~ 2019). **f** Iodine-induced thyroid risks under Scenario 2. **g** Optimal iodine provision for reducing thyroid risks under Scenario 2. **h** Scenario 3 represents groundwater as partial source in drinking water utilization with consideration of iodized salt supply in the total dietary iodine intake. **i** Iodine-induced thyroid risks under Scenario 3. **j** Optimal iodine provision for reducing thyroid risks under Scenario 3.

Integrated iodine provision is invariably necessary since daily iodine intake is jointly influenced by food, edible salt, and drinking water. Most foods have low native iodine content and contribute little to dietary intake, though marine foods ingestion could be important to a small number of countries in East Asia, North America and Europe^6^. As an auxiliary measure, staple food iodine fortification (e.g., agronomic biofortification on wheat or rice^60^) might be of potential in the short- to medium-term. Salt has a proven record as an excellent vehicle for iodine fortification to maintain world population iodine sufficiency^61^ (Fig. 6a). A relatively overlooked issue concerns iodine in drinking water, which has recently been reported as a major contributor to excess iodine intake in dozens of countries^62^. Noting that the importance of drinking water as a major iodine donor in the dietary structure, we estimate iodine-related health risks under three scenarios with varying proportions of groundwater in drinking water utilization aimed to design the optimal scheme of iodine provision. Scenario 1 denotes thyroid risks from groundwater iodine as sole source of drinking water iodine (Fig. 6b). Scenario 2 considers the thyroid risks after reducing the proportions of groundwater in drinking water utilization (Case 1: 2010 ~ 2014 water supply scheme, Supplementary Fig. 12; Case 2: 2015 ~ 2019 water supply scheme, Fig. 6e). Scenario 3 further evaluates the thyroid risks relevant to iodized salt under 2015 ~ 2019 water supply scheme, mainly considering current daily salt intake in China (Case 1: 9.3 g/day) (Supplementary Fig. 13) and that recommended by WHO (Case 2: 5.0 g/day) (Fig. 6h). As a result, thyroid risks are significantly reduced (mean R_E_ reduced from 3.13 in Scenario 1 to 1.09 ~ 1.29 in Scenario 2) by decreasing the proportion of groundwater in iodine-excess regions (Fig. 6c, f). With the promotion of iodized salt (Fig. 6f, i) in the iodine-deficient regions, thyroid risk is also greatly reduced (mean R_D_ reduced from 15.08 ~ 16.76 in Scenario 2 to 0.37 ~ 0.66 in Scenario 3). Otherwise, higher proportion of groundwater utilization in iodine-excess regions would induce greater risks (mean R_E_ 0.94 ~ 1.55 in Scenario 3) peaked in Zone II (mean R_E_ 1.15 ~ 1.76) (Fig. 6i).

The foregoing scenario analysis provides insight into optimal iodine provision (Fig. 6d, g, j) by examining the contribution of groundwater iodine in drinking water utilization at a continental scale. Without loss of generality, the non-iodized salt option is preferred in areas with groundwater iodine of 75 ~ 300 μg/L, and deiodination or alternative water sources should be considered whenever groundwater iodine exceeds 300 μg/L (see Methods), following the dietary reference values for iodine recommended by the Institute of Medicine (IOM) and the WHO^62^ (Fig. 6a). More precise countermeasures are likely to be implemented with increasing resolution of the iodine distribution. Due to the complexity of effective iodine provision, considerably more efforts are still required to meet the diverse iodine requirements of residents in different parts of the world.

In summary, we provide the national map of groundwater iodine and its species at continental scale based on a large dataset derived from 686 wells distributed in 31 provinces of China. We find that groundwater iodine is naturally controlled by the altitude, while the highest groundwater iodine appears where suffered intensive anthropogenic overexploitation. The heterogeneous distributions of both iodine and its species in groundwater significantly complicate the spatial response from their diverse health risks. Our study identifies the “hotspots” where groundwater iodine considerably contributes to population iodine nutrition level. Groundwater iodide tends to be associated with subclinical hypothyroidism particularly in higher iodine regions, while iodate impacts on thyroid risks in the presence of universal salt iodization exhibit high uncertainties in lower iodine regions. This study provides new insights into the spatial differentiation and health implication of iodine and its species, which are essential to formulating effective iodine-control strategies and sustainable groundwater management at regional, national, and global scales.

## Methods

### Monitoring wells and sample collections

To complete a national blueprint for the spatial distribution of iodine and its species in groundwater, we collected 686 groundwater samples from standard monitoring wells located in 31 provinces of China during 2016 to 2017 (Supplementary Fig. 1 and Supplementary Table 1). Sampling sites were based on China’s seven groundwater geo-environmental zones: Northeast Plain-Mountain Zone (I), Huanghuaihai-Yangtze River Delta Plain Zone (II), South China Bedrock Foothill Zone (III), Northwest Loess Plateau Zone (IV), Southwest China Karst Rock Mountain Zone (V), Northwest Arid Desert Zone (VI), and Qinghai-Tibet Plateau Alpine Frozen Soil Zone (VII). The sampling sites covered almost the whole of China including typical terrain (plain, basin, and plateau), major river basins (e.g., Yangtze, Yellow, Huai, Hai, and Pearl), and dominant urban areas (Beijing, Shanghai, Guangzhou, etc.). The layout of sampling sites was in accordance with Technical Specifications for Environmental Monitoring of Groundwater (HJ/T 164–2004)^63^. The monitoring campaign took account of drinking water sources, human interference (e.g., pollution and agricultural irrigation), and seawater intrusion.

All the 686 wells used for sampling were constructed according to China’s Regulation on Groundwater Monitoring Well Construction (DZ/T 0270–2014)^64^. 575 wells were newly constructed and 111 wells were reconstructed. Sampling conducted in the newly constructed wells was designed to reveal the baseline groundwater iodine. The reconstructed wells were used for comparison purposes, to interpret the influence of human activities.

### Groundwater sampling procedure

All groundwater sampling followed China’s standard procedure for the environmental monitoring of groundwater (HJ 494–2009)^65^. Before sample collection, each monitoring well was purged by pumping out groundwater with an outflow discharge below 100 mL/min. Outflow water quality indexes (pH, temperature, electrical conductivity, oxidation-reduction potential, dissolved oxygen, and turbidity) were measured using a portable water quality meter (WTW Multi 3630 IDS) every 5 ~ 15 min until the indexes became stable (≤ ± 10%) for three consecutive measurements^29^. Groundwater samples were then collected, filtered through a 0.45 μm membrane, and immediately stored in 250 mL iodine-free HDPE containers covered with aluminum foil to avoid light. During transportation and laboratory storage, samples were kept frozen in a refrigerator^66^.

### Groundwater chemical analysis

Alkalinity was analyzed by titration with 0.025 M HCl within 24 h after sampling. Anion contents, including chloride (Cl^−^) and sulfate radical (SO_4_^2−^), were analyzed using an ion chromatography system (IC, Thermo Fisher Scientific ICS-1100). Cations, such as potassium (K^+^), calcium (Ca^2+^), sodium (Na^+^), and magnesium (Mg^2+^), were determined using an inductively coupled plasma-optical emission spectrometer (ICP-OES, Leeman Prodigy). Ion charge imbalances were <5% for all samples. All measurements were in accordance with standard methods^67,68^ recommended by the Ministry of Ecology and Environment of China.

### Determination of iodine content and speciation

Total inorganic iodine (TII = I^−^ + IO_3_^−^) content in groundwater was determined by gas chromatography-mass spectrometry (GC-MS, Agilent 5977B GC/MSD) with derivatization, following the method proposed by Zhang. et al.^66^. In brief, for I^−^ analysis, 0.5 mL 1% acetic acid and 1 mL phosphate buffer (pH=6.5) were added to 5 mL groundwater and mixed. *N,N*-Dimethylaniline solution and 2-iodosobenzoate were then added to the mixture to oxidize I^−^ to I_2_, and form the derivative (4-iodo-*N,N*-dimethylaniline), which was measured by GC-MS after being extracted into cyclohexane solvent (with 2,4,6-tribromoaniline as the internal standard). IO_3_^−^ was estimated from the difference between I^−^ and total inorganic iodine content (TII = I^−^ + IO_3_^−^). IO_3_^−^ was first converted into I^−^ by reacting groundwater with sodium metabisulfite for 30 min (pH = 3). TII (I^−^ + IO_3_^−^) was quantified using the same procedure used for I^−^ measurement. Finally, IO_3_^−^ was calculated from the difference between I^−^ and TII.

Total iodine (TI = TII + Organo-iodine) concentration was determined by inductively coupled plasma-mass spectrometry (ICP-MS, Thermo Fisher X Series II) following Yang. et al.^69^. Organo-iodine content was calculated from the difference between TI and TII. Parallel experiments were designed for 20% groundwater samples to ensure the accuracy of experimental results, and the relative deviation in parallel samples was invariably below 20%. Detection limits were 0.012, 0.21, and 0.032 μg/L for I^−^, IO_3_^−^, and TI.

### Simulation of anthropic-induced annual groundwater storage variation

To reveal the influence of human activities on groundwater environment, the global hydrological model WaterGAP2.2d, developed by Hannes Müller Schmied et al.^35^, was used to calculate the annual variation of groundwater storage accumulation (mm) in the seven geo-environmental zones from 1971 to 2016. The approach taken was to quantify annual human use of groundwater and surface water, as well as annual water flows and water storage (with 1900 set as the zero point) based on outputs from five global water use models^70,71^, linked by the Groundwater-Surface Water Use (GWSWUSE) model, and the WaterGAP Global Hydrology Model (WGHM)^35^. With a focus on overexploitation and depletion of water resources, the simulation results properly reflect continental water storage variations. To compare groundwater storage across different geo-environmental zones, the modeled regional groundwater storage (MRWS_*i,z*_) (mm) in the *i-*th year at geo-environmental zone *z* was standardized by setting the corresponding regional simulation result in 1971 (MRWS_1971,*z*_) (mm) as datum. The normalized regional groundwater storage (NRWS_N,*i,z*_) (mm) was given by:

1 $${{{\mbox{NRWS}}}}_{i,z}={{{\mbox{MRWS}}}}_{i,z}-{{{\mbox{MRWS}}}}_{1971,z}.$$

### Health risk assessment

Non-carcinogenic risks to human of iodinated compounds were generally carried out via exposure route of oral ingestion. According to the National Health Commission of China^40,68^, the estimated iodine contribution from drinking water was about 20%. In this study, we estimate the nationwide non-carcinogenic risk of groundwater iodine by considering drinking water. Based on guidelines from the U.S. Environmental Protection Agency (USEPA)^72^, the population exposure to iodine was quantified as the following daily intake (DI) (μg/day):

2 $${{\mbox{DI}}}=\frac{{{{\mbox{C}}}}_{{{\mbox{w}}}}\times {{\mbox{I}}}{{{\mbox{R}}}}_{{{\mbox{w}}}}\times {{\mbox{EF}}}\times {{\mbox{ED}}}}{{{\mbox{BW}}}\times {{\mbox{AT}}}},$$

where C_w_ is iodine content in drinking water (μg/L), IR_w_ is drinking water ingestion rate (L/day), EF is exposure frequency (day/year), ED is exposure duration (year), BW is body weight (kg), and AT is averaging time (days). The hazard quotient (HQ) was calculated from:

3 $${{\mbox{HQ}}}=\frac{{{\mbox{DI}}}}{{{\mbox{RfD}}}},$$

where RfD is the reference dose (μg/day). In the present study, HQ represents the risk of iodine intake from groundwater.

Given that either iodine deficiency or excess could lead to severe thyroid disorders, iodine content in drinking water should obviously remain within a certain range. Owing to the absence of data on iodine RfD in the Integrated Risk Information System (IRIS) database of the USEPA, the lower and upper limits of daily iodine intake were taken to be 80 μg/day and 150 μg/day, as specified by the World Health Organization (WHO)^73^.

For non-carcinogenic risk, the allowable limit of HQ was 1. In accordance with the general rule of risk assessment, the risk of iodine deficiency (R_D_) was expressed:

4 $${{{\mbox{R}}}}_{{{\mbox{D}}}}=\frac{1}{{{{\mbox{HQ}}}}_{{{\mbox{D}}}}},$$

and the risk of iodine excess (R_E_) was given by:

5 $${{{\mbox{R}}}}_{{{\mbox{E}}}}={{{\mbox{HQ}}}}_{{{\mbox{E}}}},$$

where HQ_D_ is the iodine-deficient hazard quotient and HQ_E_ is the iodine-excess hazard quotient. Iodine-induced health risk exists when either R_D_ or R_E_ > 1. Parameters specific to the survey region played an important role in the health risk assessment. Supplementary Table 16 lists the reference values^73^ used to calculate HQ. Maps of iodine-deficient and -excess risk distribution were obtained using Kriging interpolation of HQ_D_ and HQ_E_ values.

### Ecological analyses of groundwater iodine content and epidemiological data

The surveyed data on population iodine nutritional level and thyroid diseases were obtained based on a national cross-sectional study, covering 78490 enrolled participants (aged 18 or older) from 36 sampled cities in 31 provinces of China during 2015 ~ 2017 (Supplementary Table 10, data provided by authors of a previous study^39^). Besides the correlation between population iodine nutritional level and median groundwater iodine (MGI), we also analyze the relation between prevalence of thyroid nodules (TNs)/subclinical hypothyroidism and median groundwater iodate (MGI_Iodate_)/median groundwater iodide (MGI_Iodide_) respectively.

### Contribution of groundwater iodine in drinking water iodine

Great variation exists in the proportion of groundwater used for water supply among different provinces in China. We calculated the provincial average proportion of groundwater used for water supply over a period of 5 years (2010 ~2014 and 2015 ~ 2019), from statistics provided by China’s National Bureau of Statistics (Supplementary Table 13). An index of drinking water iodine (DII) (μg/L) was determined as the product of groundwater iodine content (GIC) (μg/L) and proportion of groundwater to water supply (PGW):

6 $${{{{{\rm{DII}}}}}}={{{{{\rm{GIC}}}}}}\times {{{{{\rm{PGW}}}}}}$$

This enables a national map of the index of drinking water iodine at county level to be drawn using the Kriging method.

### Effect of drinking water and iodized salt on daily iodine intake

Daily iodine intake from drinking water (DI_W_) (μg/day) was calculated by:

7 $${{{{{{\rm{DI}}}}}}}_{{{{{{\rm{W}}}}}}}={{{{{\rm{GIC}}}}}}\times {{{{{\rm{PGW}}}}}}\times {{{{{{\rm{IR}}}}}}}_{{{{{{\rm{W}}}}}}}.$$

Daily iodine intake from iodized salt (DI_S_) (μg/day) was calculated by:

8 $${{{{{{\rm{DI}}}}}}}_{{{{{{\rm{S}}}}}}}={{{{{\rm{CI}}}}}}\times {{{{{\rm{DIS}}}}}}\times {{{{{\rm{LR}}}}}},$$

where CI is iodine content in salt (mg/kg) (Supplementary Table 17), DIS is daily intake of salt (g/day), and LR is the average loss rate of iodized salt during cooking (20%). The iodized salt and drinking water are considered to be the major sources of dietary iodine under Scenario 3. Total daily iodine intake (DI_T_) (μg/day) under Scenario 3 was determined by:

9 $${{{{{{\rm{DI}}}}}}}_{{{{{{\rm{T}}}}}}}={{{{{{\rm{DI}}}}}}}_{{{{{{\rm{S}}}}}}}+{{{{{{\rm{DI}}}}}}}_{{{{{{\rm{W}}}}}}}.$$

### Recommended limits for identifying high iodine groundwater

Recommended limits (RL) (μg/L) were proposed for determining high iodine groundwater, based on varying dietary reference values respectively defined as follows:

10 $${{\mbox{R}}}{{{\mbox{L}}}}_{{{\mbox{R}}}}=\frac{{{\mbox{RNI}}}}{{{\mbox{I}}}{{{\mbox{R}}}}_{{{\mbox{w}}}}},$$

11 $${{\mbox{R}}}{{{\mbox{L}}}}_{{{\mbox{U}}}}=\frac{{{\mbox{UL}}}}{{{\mbox{I}}}{{{\mbox{R}}}}_{{{\mbox{w}}}}},$$

where RNI is the recommended nutrient intake (μg/day) provided by WHO, and UL is the tolerable upper iodine intake level for adults (μg/day) established by the Institute of Medicine (Supplementary Table 18). Supplementary Table 19 lists the values of RL.

### Statistical analysis

The Local Moran’s Index is a local indicator of spatial association that represents significant spatial clustering of similar values about an observation^74^, and was defined as:

12 $${{{{{{\rm{I}}}}}}}_{{{{{{\rm{a}}}}}}}=\frac{{{{{{{\rm{z}}}}}}}_{{{{{{\rm{a}}}}}}}-\bar{{{{{{\rm{z}}}}}}}}{{\sigma }^{2}}{\sum }_{{{{{{\rm{b}}}}}}}\left[{{{{{{\rm{w}}}}}}}_{{{{{{\rm{ab}}}}}}}\left({{{{{{\rm{z}}}}}}}_{{{{{{\rm{b}}}}}}}-\bar{{{{{{\rm{z}}}}}}}\right)\right],$$

where z_*a*_ is the iodine content at site *a* (μg/L), z_*b*_ is the iodine content at neighboring site *b* (μg/L), w_*ab*_ is the weight of the inverse distance between observations *a* and *b*, and $\bar{{{\mbox{z}}}}$ is the average (μg/L) and σ^2^ is the variance of iodine content (μg^2^/L^2^)^74,75^.

GeoDa (Anselin, 2013) was used to calculate the Local Moran’s Index, the aim being to identify groundwater iodine-deficient or -excess areas and recognize spatial outliers, with probability value *P* < 0.05 selected as statistically significant. Iodine-deficient or -excess areas could be identified as two types of spatial clusters: High-High (a site with high iodine content in a neighborhood with high content also) and Low-Low (a site with low iodine content in a neighborhood with low content also). The remaining two types of spatial outliers were characterized as High-Low (a site with high iodine content in a neighborhood with low content) and Low-High (a site with low iodine content in a neighborhood with high content) clusters^75^.

Origin 2018 and SPSS (IBM SPSS statistics version 20.0) were utilized to evaluate correlations. All statistical tests were two-sided, with probability value *P* < 0.01 selected as statistically significant. Geospatial maps of groundwater iodine and its species were drawn using ArcGIS (ESRI ArcGIS version 10.4).

### Reporting summary

Further information on research design is available in the Nature Portfolio Reporting Summary linked to this article.

## Supplementary information

Supplementary Information

Reporting Summary

## Data Availability

All data supporting the findings of this study, including China’s national distribution maps of iodine and its species, iodine-induced health risk, and epidemiological data on thyroid diseases, are available within the paper and its supplementary information file. The geographical information of sampling sites and concentration of groundwater iodine have been deposited in the figshare database [10.6084/m9.figshare.21507528.v1]^76^. The map data used in this study is available in the GeoCloud Database developed by China Geological Survey [https://geocloud.cgs.gov.cn/].
