# Supplementary material for: Deficiency and excess of groundwater iodine and their health associations
Source: Nat Commun. 2022 Nov 29;13:7354. doi: 10.1038/s41467-022-35042-6 (PMC9708681; doi:10.1038/s41467-022-35042-6)
Supplement: Supplementary file 1 — Supplementary Information [file 41467_2022_35042_MOESM1_ESM.pdf]

Supplementary Information for:

Deficiency and excess of groundwater iodine and their health associations

Ruoqi Ma[1,2,3], Mingquan Yan[1], Peng Han[1], Ting Wang[1,4], Bin Li[1,4], Shungui Zhou[5], Tong Zheng[1], Yandi Hu[1], Alistair G. L. Borthwick[6,7], Chunmiao Zheng[8], Jinren Ni[1,2,*]

*Correspondence to: Jinren Ni, College of Environmental Sciences and Engineering, Peking University, Beijing 100871, P. R. China

E-mail addresses: jinrenni@pku.edu.cn (J.R. Ni)

This Supplementary Information contains:

- Glossary (page 2)
- Supplementary Figures 1 ~ 13 (page 3 ~ 21)
- Supplementary Tables 1 ~ 19 (page 22 ~ 49)
- Supplementary References (page 50 ~ 51)

Glossary

Reduction iodine: iodide (I^-), reduction state of iodine in the hydro-biogeochemical cycle.

Oxidation iodine: iodate (IO_3^-) and organo-iodine, oxidation state of iodine in the hydro-biogeochemical cycle.

Porewater: water stored in the compressible sediments, which could be released into adjacent aquifers in the process of sediment compaction.

Iodine enrichment ratio: an index used to evaluate the level of iodine released from the solid to the aqueous phase, and defined as iodide/chloride (I^-/Cl^-) in groundwater.

Risk index of iodine deficiency (R_D): evaluation index of non-carcinogenic risks response to iodine deficiency, which is equal numerically to the reciprocal of iodine-deficient hazard quotient.

Risk index of iodine excess (R_E): evaluation index of non-carcinogenic risks response to iodine excess, which is equal numerically to the iodine-excess hazard quotient.

Supplementary Figures

Supplementary Fig. 1 | Sampling sites of groundwater in 686 monitoring wells throughout China

Supplementary Fig. 2 | Distribution characteristics of iodine in groundwater throughout China

Supplementary Fig. 3 | Time series of annual groundwater storage accumulation (1971 ~ 2016) in seven geo-environmental zones of China

Supplementary Fig. 4 | Summary of previous conceptual models for iodine enrichment and transformation in groundwater

Supplementary Fig. 5 | Spatial distribution of total iodine content and its species in groundwater throughout China

Supplementary Fig. 6 | Distribution characteristics of groundwater iodine species

Supplementary Fig. 7 | Relationships between prevalence of thyroid diseases and iodine nutrient levels

Supplementary Fig. 8 | Comparison of daily iodine intake from drinking water and iodized salt

Supplementary Fig. 9 | Health risks posed by total groundwater iodine in seven geo-environmental zones

Supplementary Fig. 10 | Distribution of drinking water iodine based on the national survey data from National Health Commission of P.R. China

Supplementary Fig. 11 | Spatial response of thyroid risks to groundwater iodine speciation at provincial level in China

Supplementary Fig. 12 | Optimal iodine provision in Scenario 2 (Case 1)

Supplementary Fig. 13 | Optimal iodine provision in Scenario 3 (Case 1)

53 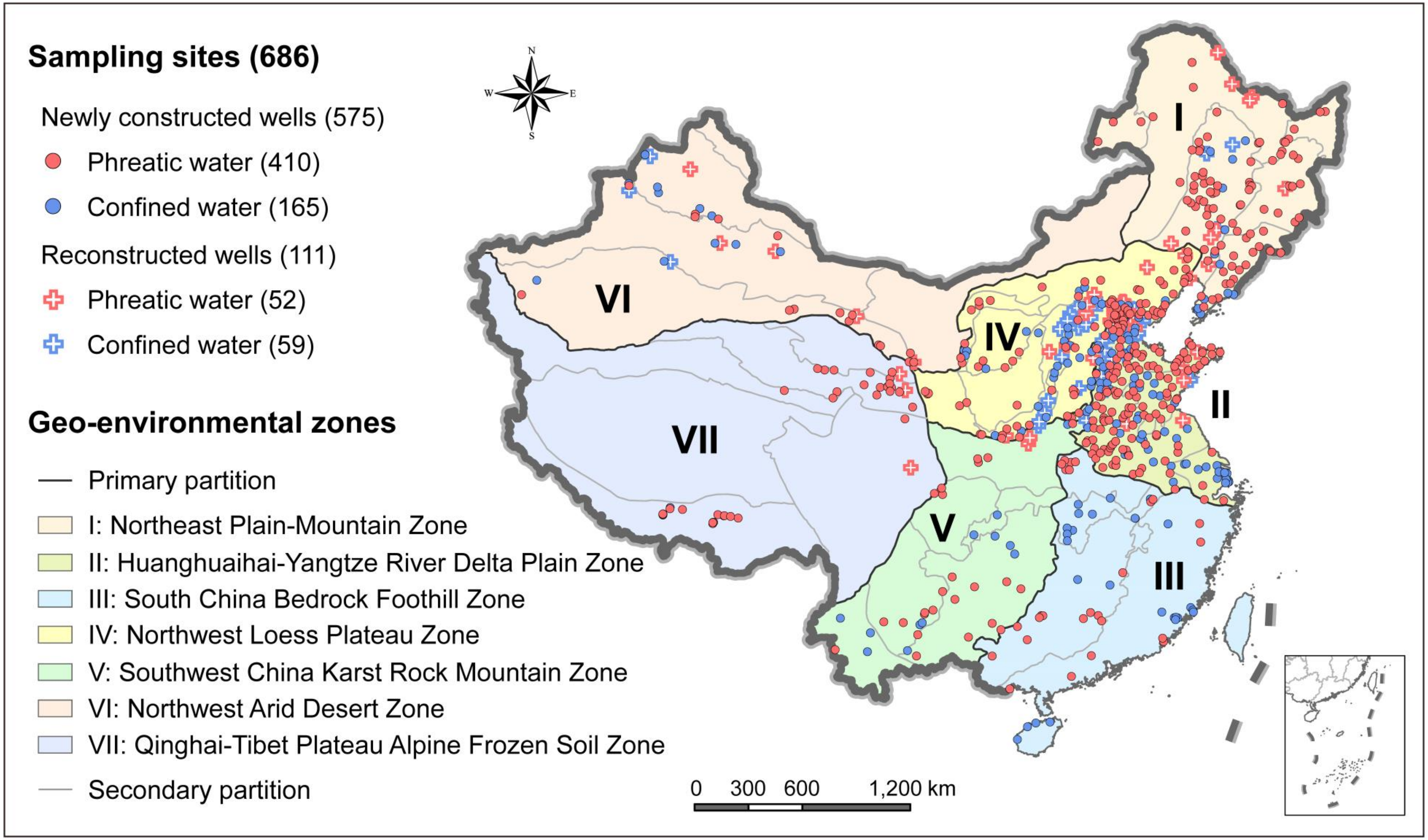
Sampling sites (686)
Newly constructed wells (575)
Phreatic water (410)
Confined water (165)
Reconstructed wells (111)
Phreatic water (52)
Confined water (59)
Geo-environmental zones
Primary partition
I: Northeast Plain-Mountain Zone
II: Huanghuaihai-Yangtze River Delta Plain Zone
III: South China Bedrock Foothill Zone
IV: Northwest Loess Plateau Zone
V: Southwest China Karst Rock Mountain Zone
VI: Northwest Arid Desert Zone
VII: Qinghai-Tibet Plateau Alpine Frozen Soil Zone
Secondary partition
I
II
III
IV
V
VI
VII
N
S
W
E
0 300 600 1,200 km

Supplementary Fig. 1 | Sampling sites of groundwater in 686 monitoring wells throughout China. Circular and hollow crosses indicate samples from newly constructed and reconstructed wells. Red and blue dots represent phreatic and confined water samples. China’s geo-environmental zones are shaded in different colors on national map, namely: Northeast Plain-Mountain Zone (I), Huanghuaihai-Yangtze River Delta Plain Zone (II), South China Bedrock Foothill Zone (III), Northwest Loess Plateau Zone (IV), Southwest China Karst Rock Mountain Zone (V), Northwest Arid Desert Zone (VI), and Qinghai-Tibet Plateau Alpine Frozen Soil Zone (VII).

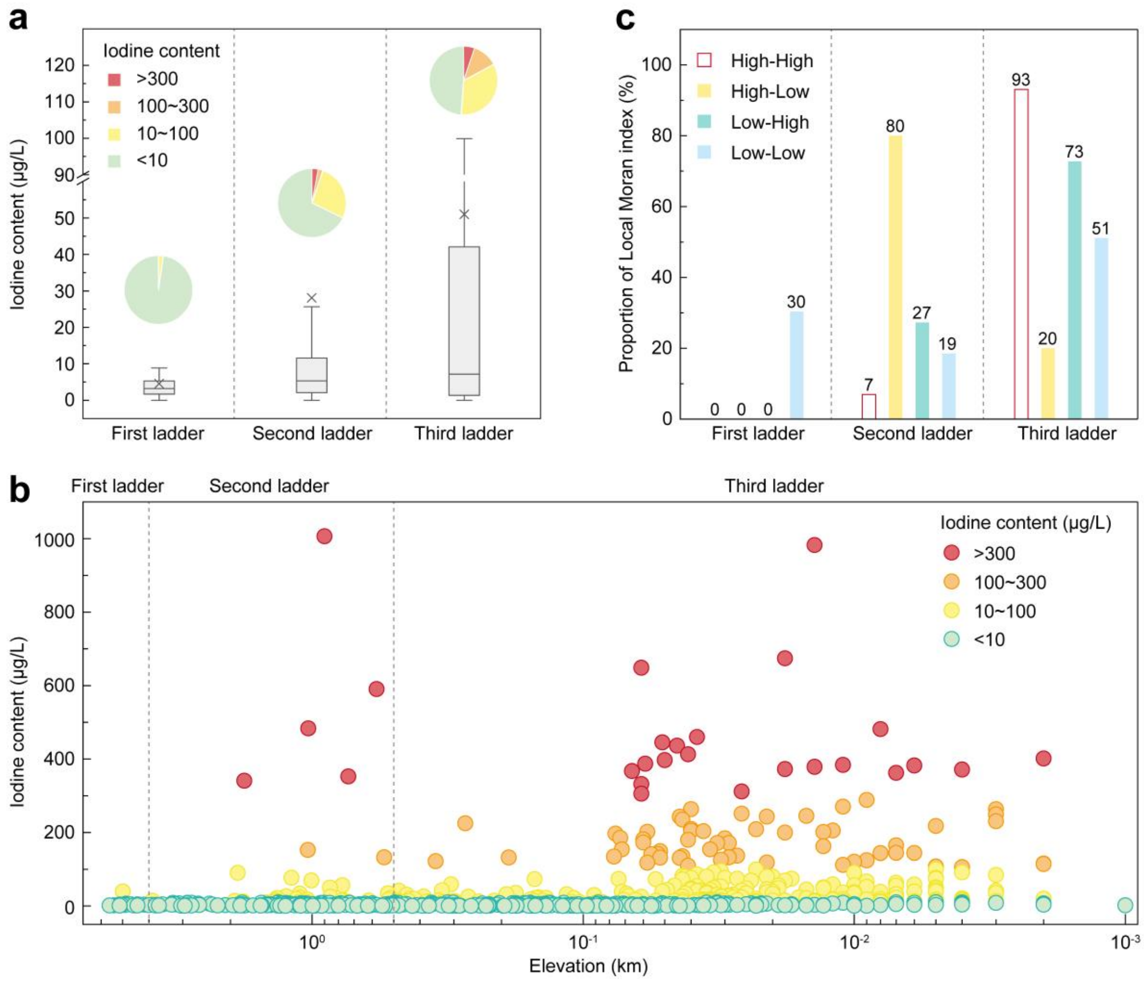

Supplementary Fig. 2 | Distribution characteristics of iodine in groundwater throughout China. **a**, Pie charts and box plots indicating distribution of groundwater iodine content in three topographic ladders of China (n = 686). The box is bounded by the first and the third quartile with a horizontal line at the median and a cross at the mean, and whiskers extend to the maximum and minimum value in 1.5 times interquartile range. **b**, Variation in groundwater iodine content with elevation above mean sea level. **c**, Histograms showing proportions of different Local Moran Indexes (with 1200 km threshold distance) in three topographic ladders.

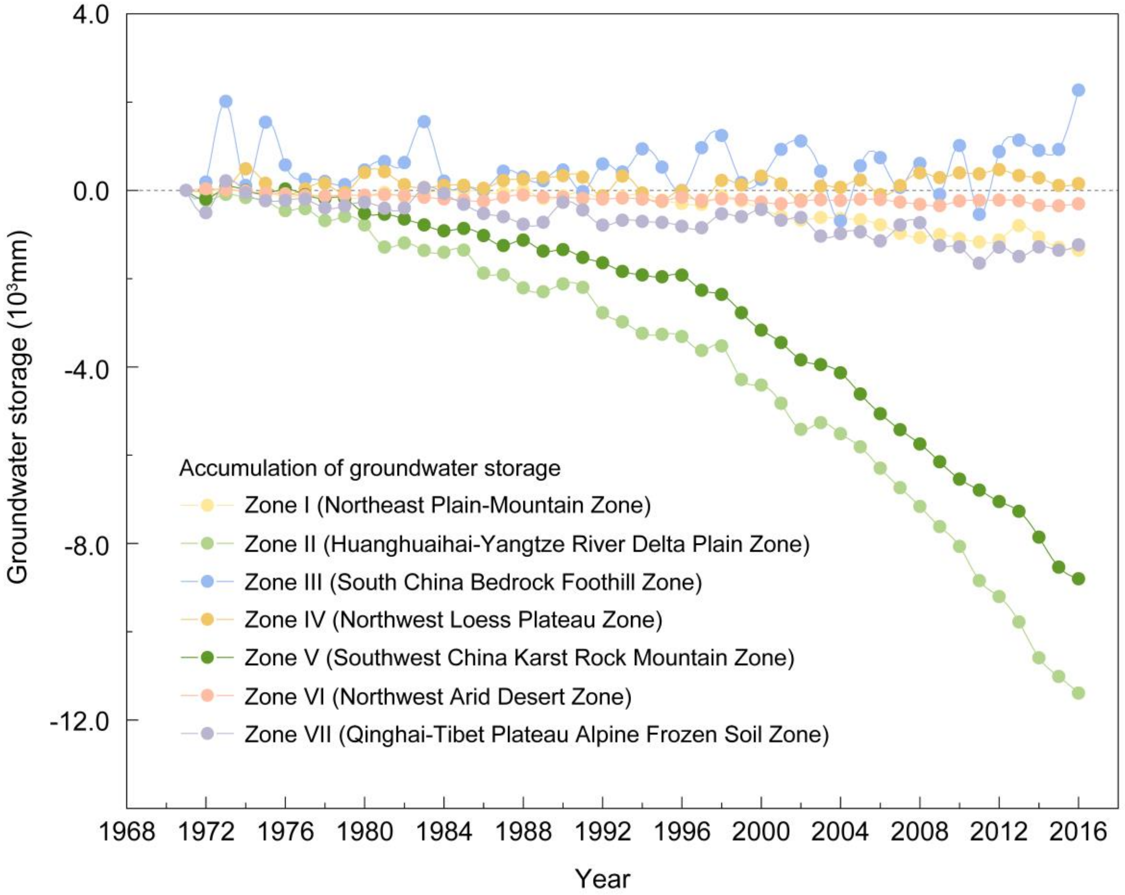

Supplementary Fig. 3 | Time series of annual groundwater storage accumulation (1971 ~ 2016) in seven geo-environmental zones of China.

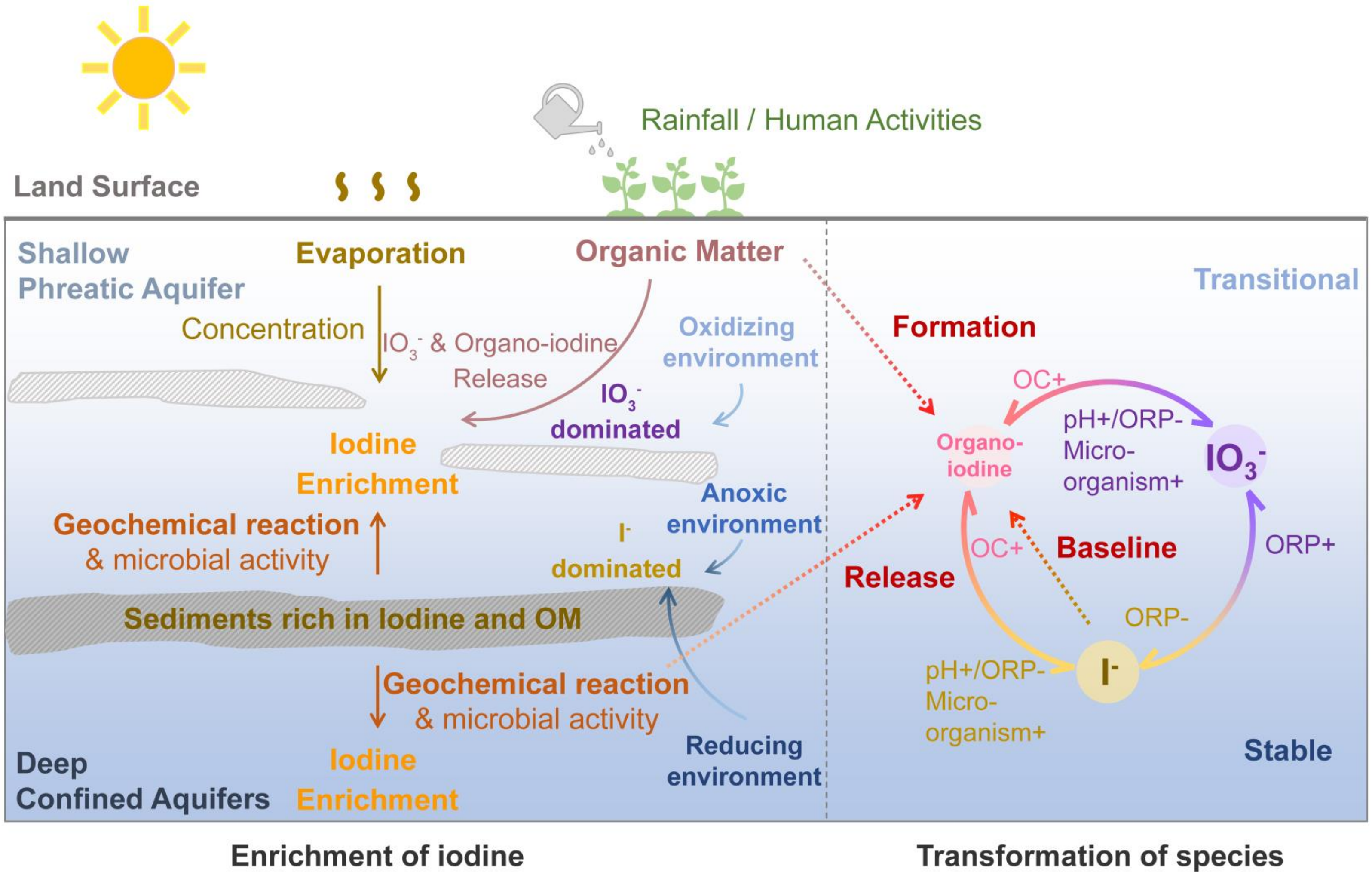

70

71 **Supplementary Fig. 4 | Summary of previous conceptual models for iodine enrichment and transformation in groundwater[1-5].**

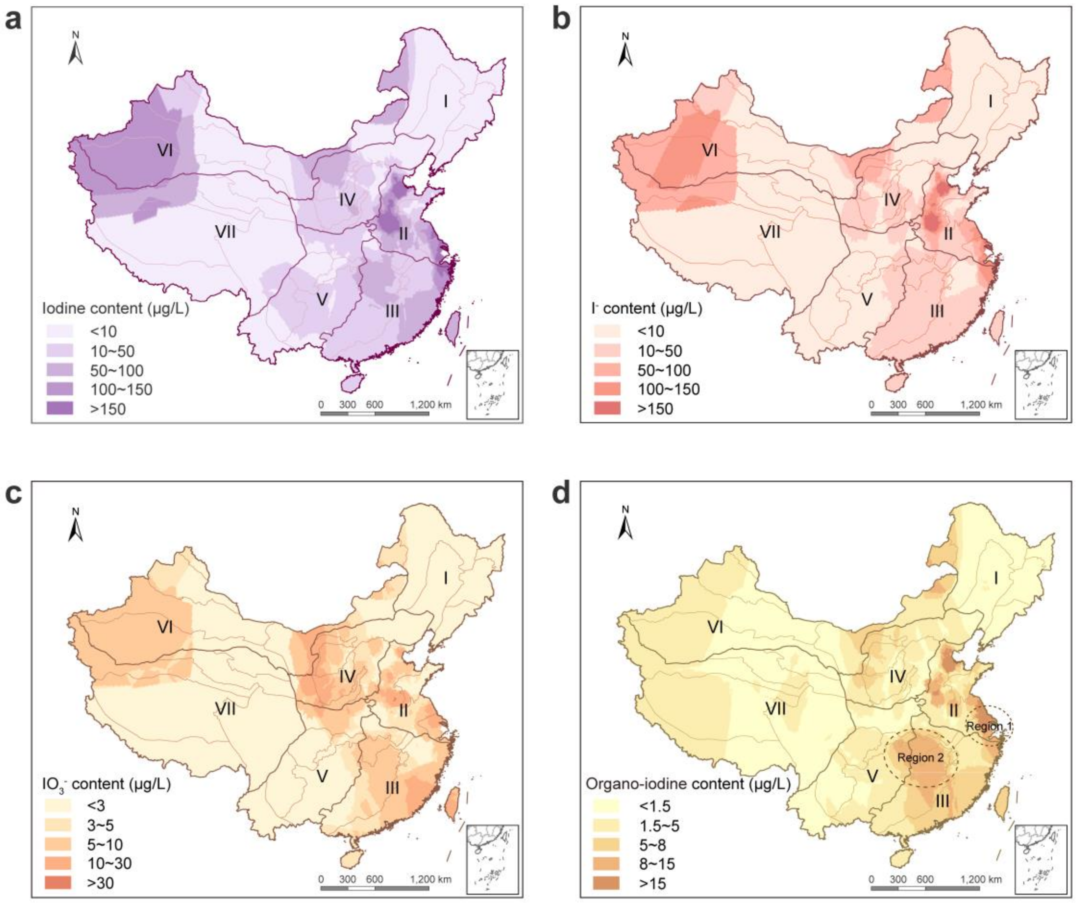

Supplementary Fig. 5 | Spatial distribution of total iodine content and its species in groundwater throughout China. a, Total iodine. **b**, Iodide. **c**, Iodate. **d**, Organo-iodine. Regions marked by dotted lines in (**d**) are the Yangtze River Delta Plain (Region 1) and the Lianghu Plain (Region 2).

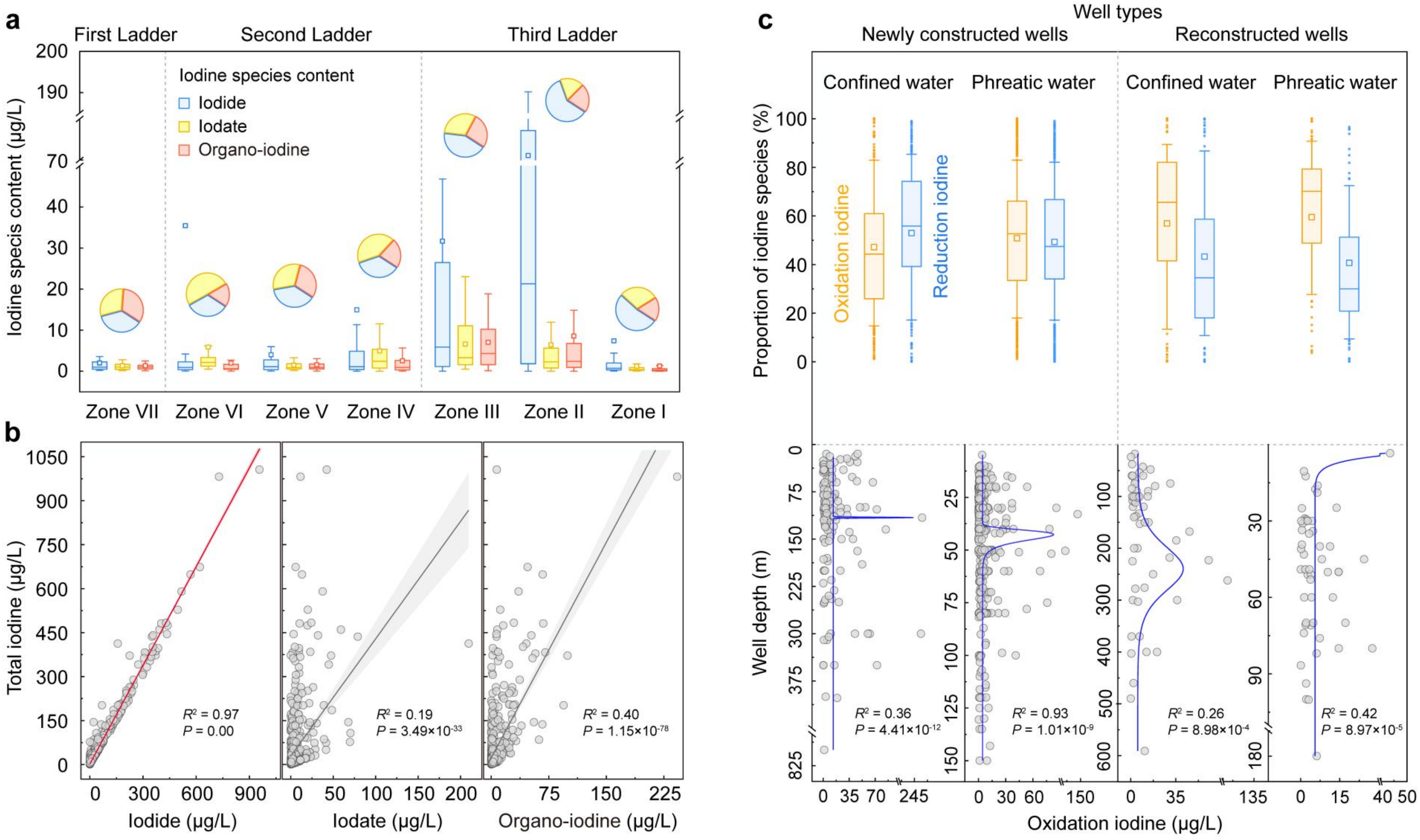

a
First Ladder
Second Ladder
Third Ladder
Iodine species content
Iodide
Iodate
Organo-iodine
Iodine specis content (μg/L)
Zone VII
Zone VI
Zone V
Zone IV
Zone III
Zone II
Zone I
b
Total iodine (μg/L)
R^2 = 0.97
P = 0.00
R^2 = 0.19
P = 3.49×10^{-33}
R^2 = 0.40
P = 1.15×10^{-78}
Iodide (μg/L)
Iodate (μg/L)
Organo-iodine (μg/L)
c
Well types
Newly constructed wells
Reconstructed wells
Confined water
Phreatic water
Confined water
Phreatic water
Proportion of iodine species (%)
Oxidation iodine
Reduction iodine
Well depth (m)
R^2 = 0.36
P = 4.41×10^{-12}
R^2 = 0.93
P = 1.01×10^{-9}
R^2 = 0.26
P = 8.98×10^{-4}
R^2 = 0.42
P = 8.97×10^{-5}
Oxidation iodine (μg/L)

Supplementary Fig. 6 | Distribution characteristics of groundwater iodine species. a, Pie charts and box plots indicating distribution of iodine species (I^-, IO_3^- and organo-iodine) in the seven geo-environmental zones of China (n = 686). The box is bounded by the first and the third quartile with a horizontal line at the median and a hollow square at the mean, and whiskers extend to the maximum and minimum value in 1.5 times interquartile range. **b**, Correlations between three iodine species and total iodine content (two-sided *t*-test). The pink line (or shade) and grey lines (or shades) are regression lines (or the 95% prediction intervals) of total iodine and different iodine species contents. **c**, Proportions of iodine species (I^-: reduction iodine, IO_3^- and organo-iodine: oxidation iodine) and variations in oxidation iodine content with well depth for different types of groundwater (n = 686, two-sided *F*-test). The box is bounded by the 35% and the 65% quartile with a horizontal line at the median and a hollow square at the mean, and whiskers extend to the maximum and minimum value in the range of 20% to 80% quartile.

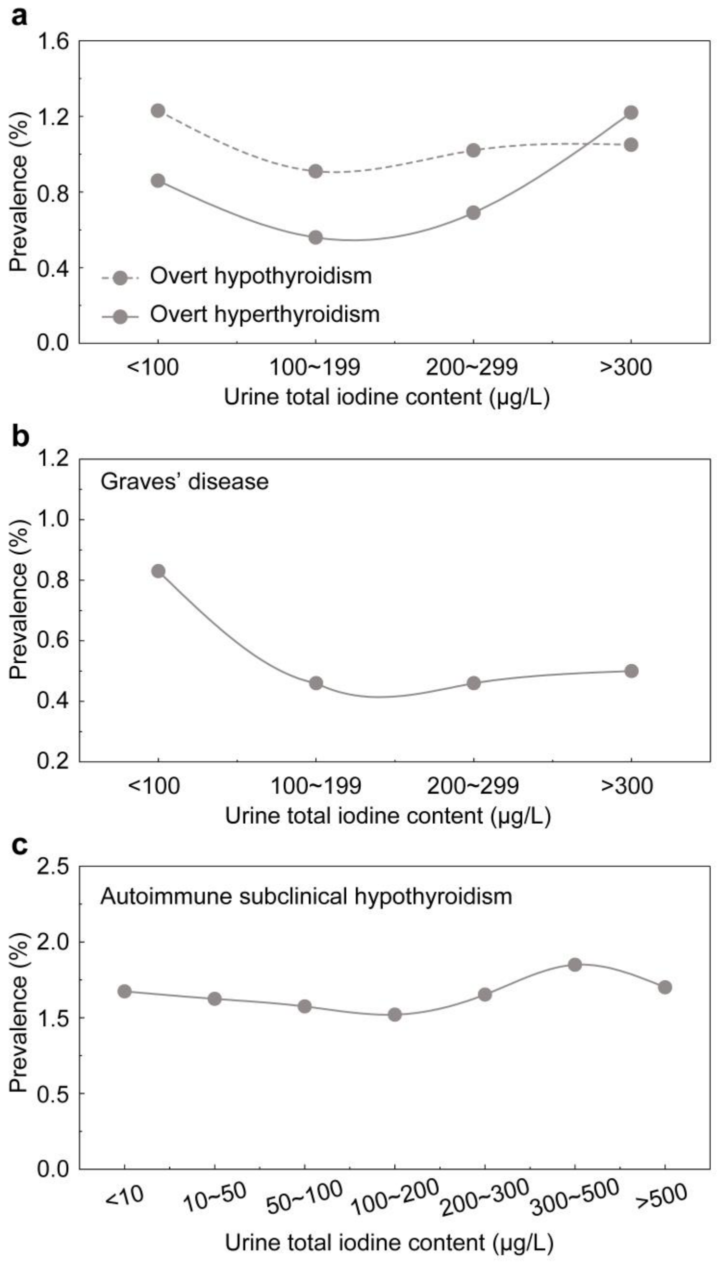

Supplementary Fig. 7 | Relationships between prevalence of thyroid diseases and iodine nutrient levels[6]. a, Prevalence of overt hypothyroidism and hyperthyroidism. **b**, Prevalence of Graves' disease. **c**, Prevalence of autoimmune subclinical hypothyroidism.

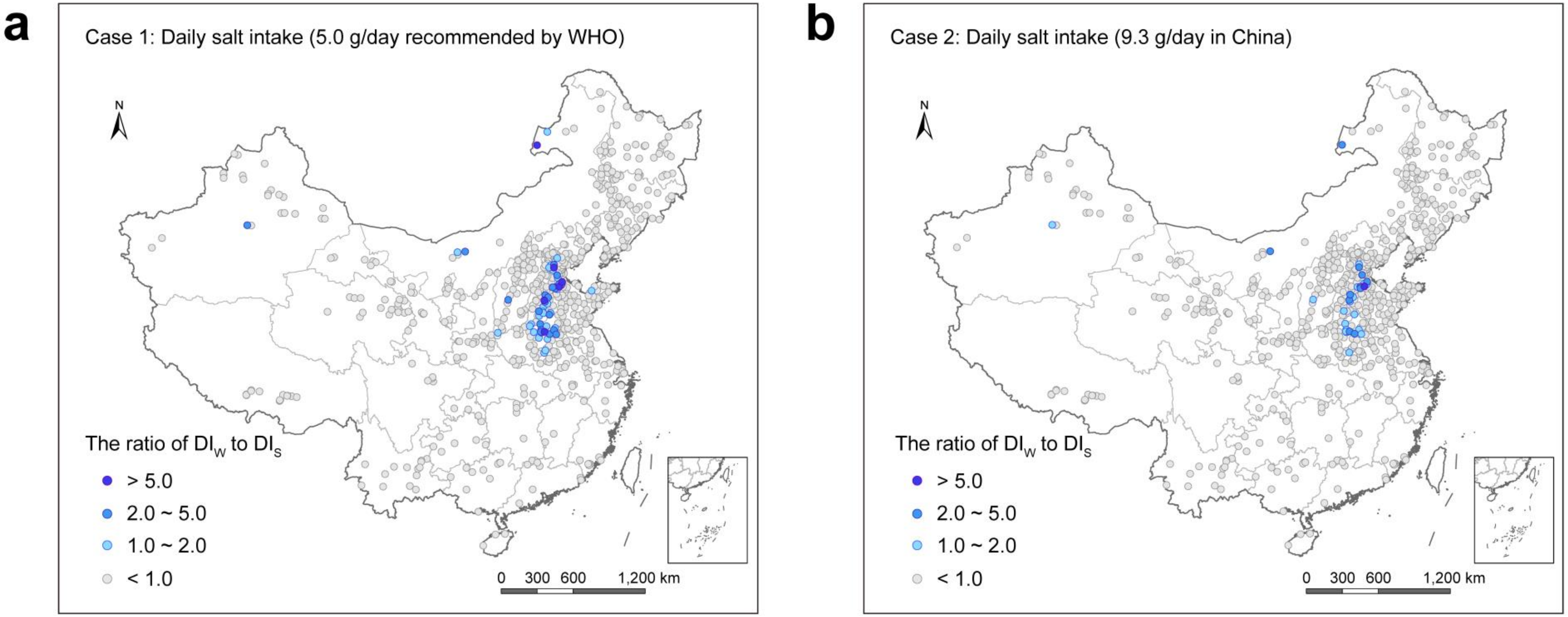

Supplementary Fig. 8 | Comparison of daily iodine intake from drinking water and iodized salt. a, Case 1 represents daily salt intake (5.0 g/day) recommended by World Health Organization (WHO). **b**, Case 2 represents daily salt intake (9.3 g/day) in China.

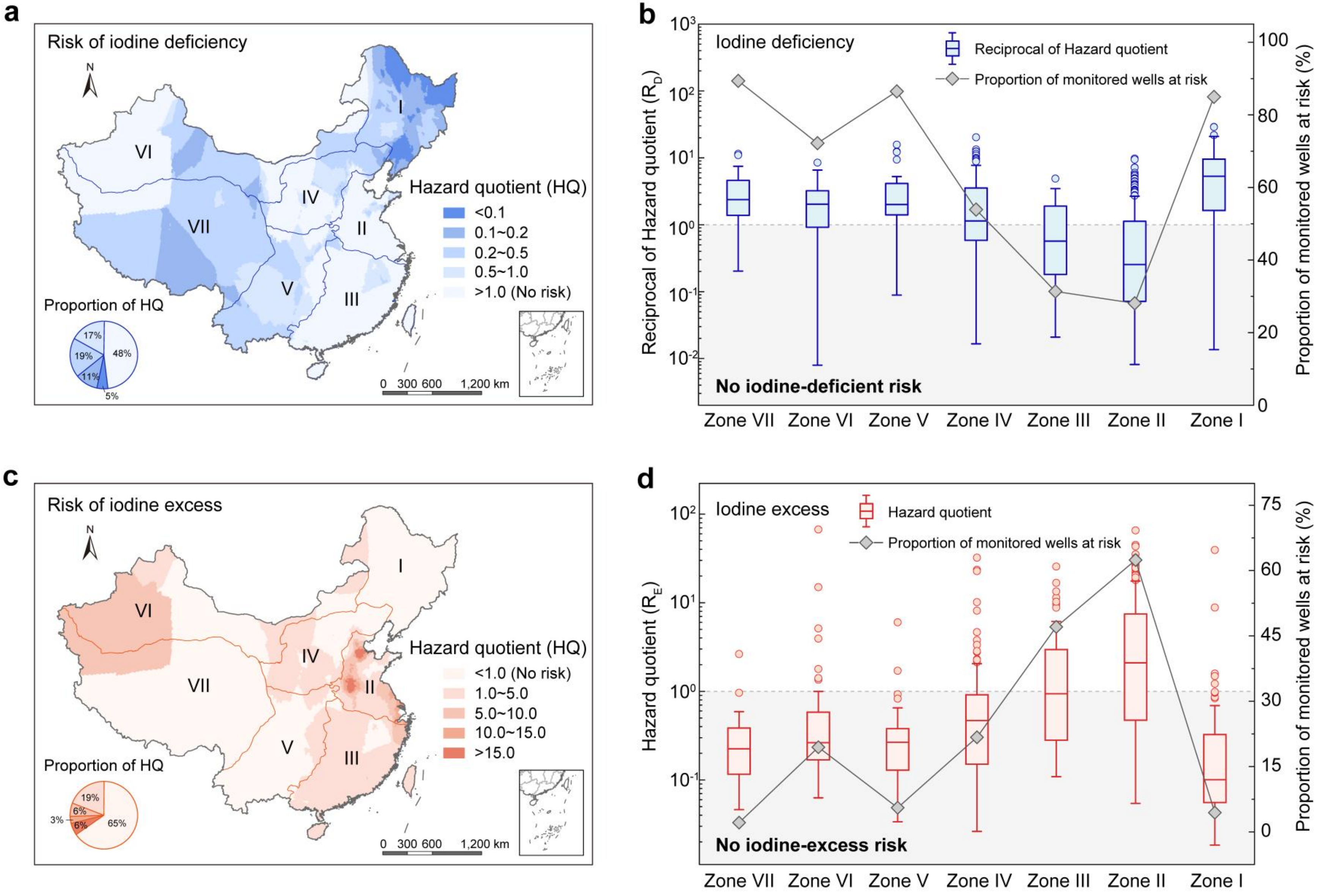

a
Risk of iodine deficiency
N
I
II
III
IV
V
VI
VII
Hazard quotient (HQ)
<0.1
0.1~0.2
0.2~0.5
0.5~1.0
>1.0 (No risk)
Proportion of HQ
17%
19%
11%
5%
48%
0 300 600 1,200 km
b
Iodine deficiency
Reciprocal of Hazard quotient
Proportion of monitored wells at risk
Reciprocal of Hazard quotient (R_D)
Proportion of monitored wells at risk (%)
No iodine-deficient risk
Zone VII
Zone VI
Zone V
Zone IV
Zone III
Zone II
Zone I
c
Risk of iodine excess
Hazard quotient (HQ)
<1.0 (No risk)
1.0~5.0
5.0~10.0
10.0~15.0
>15.0
Proportion of HQ
19%
6%
3%
6%
65%
d
Iodine excess
Hazard quotient
Proportion of monitored wells at risk
Hazard quotient (R_E)
No iodine-excess risk

Supplementary Fig. 9 | Health risks posed by total groundwater iodine in seven geo-environmental zones. a, Spatial distribution of iodine-deficient hazard quotient throughout China. **b**, Box plots showing groundwater iodine deficiency risk in the seven geo-environmental zones, expressed using the reciprocal of hazard quotient (R_D) (n = 686). **c**, Spatial distribution of iodine-excess hazard quotient. **d**, Box plots showing groundwater iodine excess risk (R_E) in the seven geo-environmental zones (n = 686). Gray areas in (**b**) and (**d**) represent no iodine deficiency or excess risk. The box in (**b**) and (**d**) is bounded by the first and the third quartile with a horizontal line at the median, and whiskers extend to the maximum and minimum value in 1.5 times interquartile range.

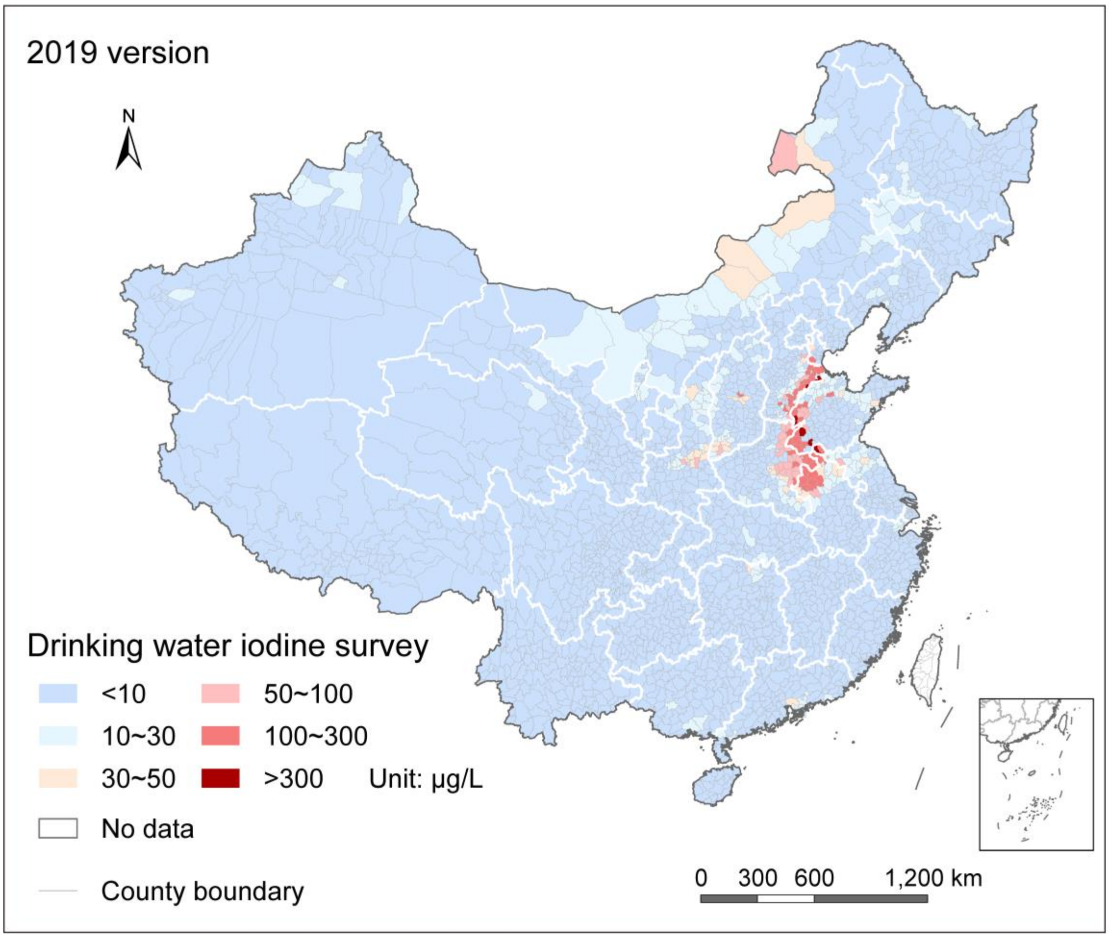

Supplementary Fig. 10 | Distribution of drinking water iodine based on the national survey data from National Health Commission of P.R. China[7].

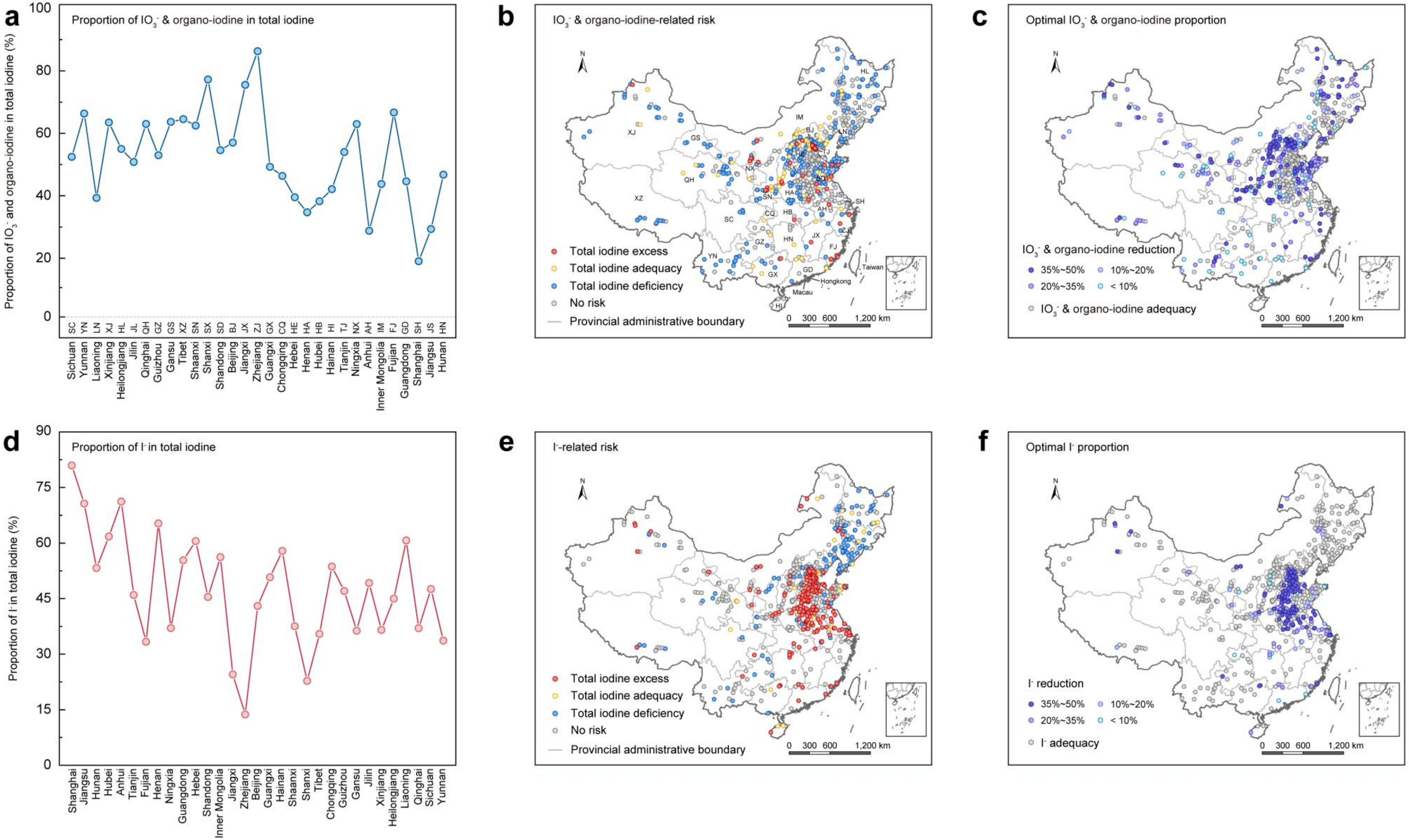

a
Proportion of IO_3^- & organo-iodine in total iodine
Proportion of IO_3^- and organo-iodine in total iodine (%)
100
80
60
40
20
0
SC YN LN XJ HL JL QH GZ GS XZ SN SX SD BJ JX ZJ GX CQ HE HA HB HI TJ NX AH IM FJ GD SH JS HN
Sichuan Yunnan Liaoning Xinjiang Heilongjiang Jilin Qinghai Guizhou Gansu Tibet Shaanxi Shanxi Shandong Beijing Jiangxi Zhejiang Guangxi Chongqing Hebei Henan Hubei Hainan Tianjin Ningxia Anhui Inner Mongolia Fujian Guangdong Shanghai Jiangsu Hunan
b
IO_3^- & organo-iodine-related risk
N
XJ
XZ
GS
QH
NX
IM
BJ
TJ
HL
JL
LN
HE
SX
SD
SN
HA
JS
SH
AH
HB
CQ
SC
GZ
HN
JX
ZJ
FJ
YN
GX
GD
Taiwan
Hongkong
Macau
HI
Total iodine excess
Total iodine adequacy
Total iodine deficiency
No risk
Provincial administrative boundary
0 300 600 1,200 km
c
Optimal IO_3^- & organo-iodine proportion
N
IO_3^- & organo-iodine reduction
35%~50%
10%~20%
20%~35%
< 10%
IO_3^- & organo-iodine adequacy
0 300 600 1,200 km
d
Proportion of I^- in total iodine
Proportion of I^- in total iodine (%)
90
75
60
45
30
15
0
Shanghai Jiangsu Hunan Hubei Anhui Tianjin Fujian Henan Ningxia Guangdong Hebei Shandong Inner Mongolia Jiangxi Zhejiang Beijing Guangxi Hainan Shaanxi Shanxi Tibet Chongqing Guizhou Gansu Jilin Xinjiang Heilongjiang Liaoning Qinghai Sichuan Yunnan
e
I^--related risk
N
Total iodine excess
Total iodine adequacy
Total iodine deficiency
No risk
Provincial administrative boundary
0 300 600 1,200 km
f
Optimal I^- proportion
N
I^- reduction
35%~50%
10%~20%
20%~35%
< 10%
I^- adequacy
0 300 600 1,200 km

Supplementary Fig. 11 | Spatial response of thyroid risks to groundwater iodine speciation at provincial level in China. **a**, Proportions of oxidation iodine (IO_3^- and organo-iodine) in total iodine at provincial level. **b**, Thyroid risks response to IO_3^- and organo-iodine in groundwater iodine (with proportion of IO_3^- and organo-iodine in total iodine > 50%). **c**, Optimal IO_3^- and organo-iodine proportion corresponding to tolerable thyroid risks. **d**, Proportions of reduction iodine (I^-) in total iodine at provincial level. **e**, Thyroid risks response to I^- in groundwater iodine (with proportion of I^- in total iodine > 50%). **f**, Optimal I^- proportion corresponding to tolerable thyroid risks.

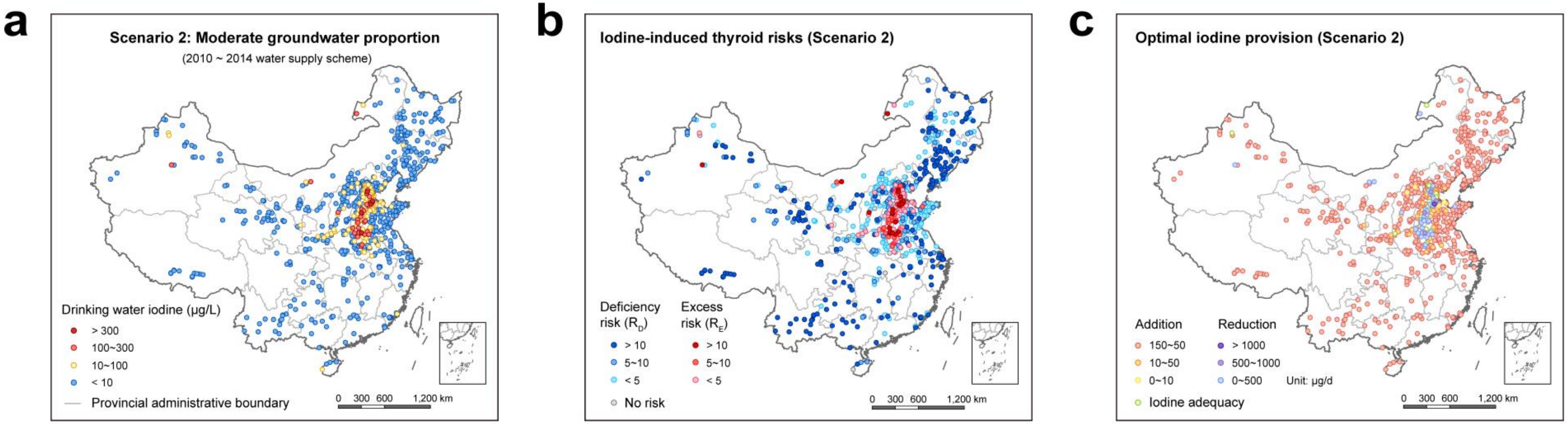

Supplementary Fig. 12 | Optimal iodine provision in Scenario 2 (Case 1). a, Scenario 2 represents groundwater as a partial source in drinking water utilization according to the water supply scheme (2010 ~ 2014). **b**, Iodine-induced thyroid risks under Scenario 2. **c**, Optimal iodine provision for reducing thyroid risks under Scenario 2.

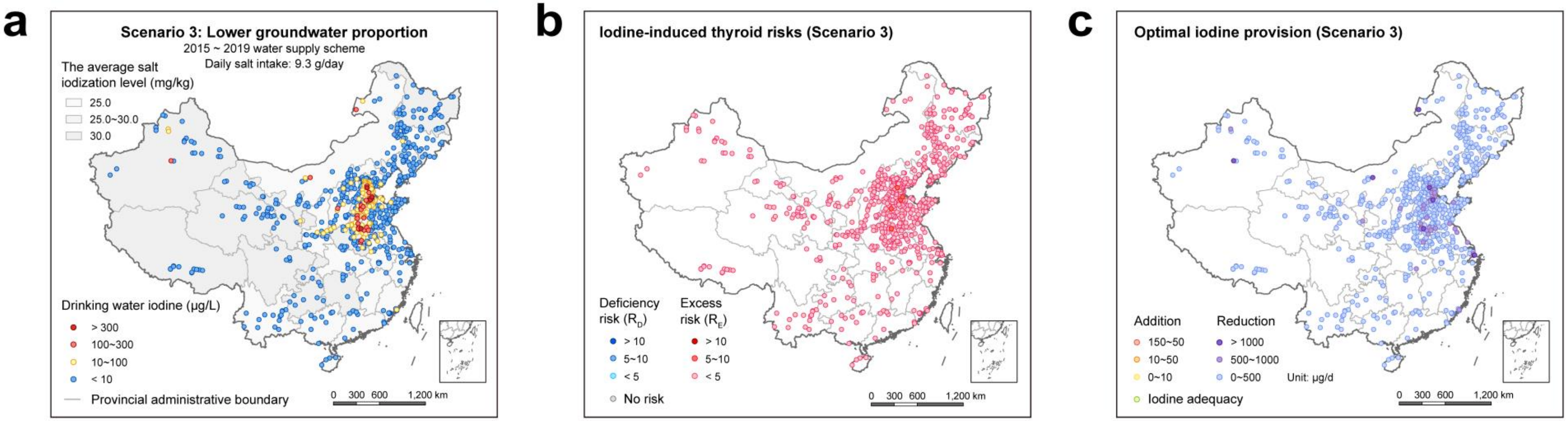

Supplementary Fig. 13 | Optimal iodine provision in Scenario 3 (Case 1). a, Scenario 3 represents groundwater as a partial source in drinking water utilization with consideration of iodized salt supply (daily salt intake 9.3 g/day) in the total dietary iodine intake. **b**, Iodine-induced thyroid risks under Scenario 3. **c**, Optimal iodine provision for reducing thyroid risks under Scenario 3.

Supplementary Tables

Supplementary Table 1 | Information on groundwater sampling sites

Supplementary Table 2 | Contents of groundwater iodine and its species in different geo-environmental zones of China

Supplementary Table 3 | Hydrochemistry composition of groundwater in Zones I and II

Supplementary Table 4 | Temporal variation of groundwater storage accumulation in Zones I and II (1971 ~ 2016)

Supplementary Table 5 | Annual water supply volume ($\times 10^8$ m^3) of main provinces involved in Zone I

Supplementary Table 6 | Annual water supply volume ($\times 10^8$ m^3) of main provinces involved in Zone II

Supplementary Table 7 | Annual groundwater resource volumes ($\times 10^8$ m^3) of main provinces involved in Zone I and Zone II

Supplementary Table 8 | Proportions of iodine species in different groundwater types

Supplementary Table 9 | Mean and 95th percentile values of hazard quotient (HQ) via drinking water consumption

Supplementary Table 10 | Iodine state and thyroid nodules prevalence in 36 cities from 31 provinces of China

Supplementary Table 11 | Proportion of monitored wells at iodine-induced risk at provincial level in China

Supplementary Table 12 | Median groundwater iodine content at provincial level in China

Supplementary Table 13 | Average proportions of groundwater used for water supply over 5 years (2010 ~ 2014 and 2015 ~ 2019) at provincial level in China

Supplementary Table 14 | Iodine content in drinking water of China

Supplementary Table 15 | Proportion of groundwater iodine species at provincial level in China

Supplementary Table 16 | Key parameters for iodine-induced non-carcinogenic risk assessment

Supplementary Table 17 | Salt iodization levels in different provinces of China

Supplementary Table 18 | Dietary reference values for iodine

Supplementary Table 19 | Recommended limits for identifying high iodine groundwater

Supplementary Table 1 | Information on groundwater sampling sites.

Geo-environmental zone	Terrain partition	Well type	Groundwater type	Number of samples
Northeast plain-mountain (Zone I)	III	Newly constructed	Phreatic	88
			Confined	13
		Reconstructed	Phreatic	10
			Confined	3
Huanghuaihai-Yangtze river delta plain (Zone II)	III	Newly constructed	Phreatic	156
			Confined	72
		Reconstructed	Phreatic	22
			Confined	35
South China bedrock low mountain foothill (Zone III)	III	Newly constructed	Phreatic	26
			Confined	25
		Reconstructed	Phreatic	0
			Confined	0

Supplementary Table 1 | Information on groundwater sampling sites (*continued*).

Geo-environmental zone	Terrain partition	Well type	Groundwater type	Number of samples
Northwest loess plateau (Zone IV)	II	Newly constructed	Phreatic	59
			Confined	29
		Reconstructed	Phreatic	10
			Confined	18
Southwest China Karst rock mountain (Zone V)	II	Newly constructed	Phreatic	24
			Confined	11
		Reconstructed	Phreatic	2
			Confined	0
Northwest arid desert (Zone VI)	II	Newly constructed	Phreatic	17
			Confined	11
		Reconstructed	Phreatic	5
			Confined	3
Qinghai-Tibet plateau Alpine frozen soil areas (Zone VII)	I	Newly constructed	Phreatic	40
			Confined	4
		Reconstructed	Phreatic	3
			Confined	0

Supplementary Table 2 | Contents of groundwater iodine and its species in different geo-environmental zones of China.

Geo-environmental zone	Content (μg/L)		Median (μg/L)	Mean (μg/L)
Zone I	Total iodine	0.28~590.40	1.51	9.85
	Iodide	0.01~517.14	0.69	7.38
	Iodate	<DL~37.64	0.40	1.21
	Organo-iodine	0.00~39.37	0.23	1.25
Zone II	Total iodine	0.82~982.30	31.50	86.09
	Iodide	0.01~728.52	21.30	71.10
	Iodate	<DL~209.59	2.29	6.42
	Organo-iodine	0.00~242.27	2.38	8.56
Zone III	Total iodine	1.63~384.10	14.08	45.33
	Iodide	0.01~352.19	5.88	31.67
	Iodate	0.46~27.91	3.31	6.60
	Organo-iodine	0.12~28.90	4.31	7.06
Zone IV	Total iodine	0.39~483.60	7.02	22.37
	Iodide	0.01~429.98	1.08	14.93
	Iodate	<DL~44.82	2.41	4.92
	Organo-iodine	0.00~29.43	0.90	2.53
Zone V	Total iodine	0.51~90.20	4.00	7.03
	Iodide	0.02~79.21	1.08	4.00
	Iodate	<DL~10.28	0.88	1.48
	Organo-iodine	0.02~9.87	0.88	1.55

DL = detection limit.

Supplementary Table 2 | Contents of groundwater iodine and its species in different geo-environmental zones of China (*continued*).

Geo-environmental zone	Content (µg/L)		Median (µg/L)	Mean (µg/L)
Zone VI	Total iodine	0.94~1006.25	3.96	43.26
	Iodide	0.01~956.73	0.91	35.43
	Iodate	0.48~69.73	2.12	5.87
	Organo-iodine	0.01~16.37	0.59	1.96
Zone VII	Total iodine	0.69~39.56	3.38	4.67
	Iodide	0.18~23.93	0.98	2.00
	Iodate	<DL~4.96	1.02	1.32
	Organo-iodine	0.10~10.96	1.02	1.34

DL = detection limit.

Supplementary Table 3 | Hydrochemistry composition of groundwater in Zones I and II.

Index	Units	Minimum		Maximum		Median		Standard deviation		DL
		Zone I	Zone II	Zone I	Zone II	Zone I	Zone II	Zone I	Zone II	
K^+	mg/L	<DL	<DL	95.00	132.00	1.3	1.30	9.03	12.03	0.03
Na^+	mg/L	<DL	3.99	970.00	7421.00	30.26	105.54	121.55	623.53	0.10
Ca^{2+}	mg/L	1.61	1.82	245.03	748.59	45.49	69.90	40.98	77.85	1.00
Mg^{2+}	mg/L	2.51	<DL	145.54	956.00	18.06	29.45	21.95	101.78	1.00
CO_3^{2-}	mg/L	<DL	<DL	1.75	145.00	<DL	<DL	0.17	12.91	-
HCO_3^-	mg/L	<DL	<DL	25.11	1677.00	3.18	387.00	4.05	217.22	5.00
Cl^-	mg/L	<DL	<DL	1017.10	10115.46	14.08	61.01	119.82	790.00	0.04
SO_4^{2-}	mg/L	<DL	<DL	1002.80	1970.27	14.98	72.29	111.28	307.79	0.10

DL = detection limit.

Supplementary Table 4 | Temporal variation of groundwater storage accumulation [a] in Zones I and II (1971 ~ 2016).

Year	Groundwater storage accumulation (mm)		Year	Groundwater storage accumulation (mm)	
	Zone I	Zone II		Zone I	Zone II
1971	0.0	0.0	1983	-50.6	-1359.3
1972	-33.4	-226.2	1984	-21.3	-1403.1
1973	23.0	-93.3	1985	0.3	-1354.7
1974	-47.1	-165.9	1986	-31.6	-1877.2
1975	-50.0	-236.3	1987	-62.4	-1914.5
1976	-145.6	-460.1	1988	12.4	-2211.8
1977	-106.6	-416.2	1989	-188.0	-2297.7
1978	-135.1	-684.9	1990	-129.8	-2119.3
1979	-181.7	-590.2	1991	-49.2	-2198.4
1980	-147.8	-786.1	1992	-184.2	-2773.3
1981	-51.6	-1283.8	1993	-151.0	-2980.2
1982	-150.5	-1195.2	1994	-82.7	-3241.0

[a] Groundwater storage accumulation: the accumulated depth (mm) of groundwater per unit area.

Supplementary Table 4 | Temporal variation of groundwater storage accumulation [a] in Zones I and II (1971 ~ 2016) (*continued*).

Year	Groundwater storage accumulation (mm)		Year	Groundwater storage accumulation (mm)	
	Zone I	Zone II		Zone I	Zone II
1995	-220.2	-3261.5	2006	-780.2	-6295.8
1996	-296.6	-3311.0	2007	-972.4	-6738.4
1997	-316.7	-3628.0	2008	-1069.4	-7161.7
1998	-134.0	-3525.6	2009	-1004.9	-7619.4
1999	-239.6	-4287.6	2010	-1093.0	-8065.6
2000	-419.3	-4411.9	2011	-1169.4	-8841.6
2001	-587.8	-4823.3	2012	-1126.8	-9201.7
2002	-673.7	-5415.2	2013	-800.9	-9777.4
2003	-617.6	-5264.1	2014	-1062.5	-10591.2
2004	-639.6	-5513.9	2015	-1291.9	-11012.2
2005	-668.8	-5810.7	2016	-1358.0	-11389.4

[a] Groundwater storage accumulation: the accumulated depth (mm) of groundwater per unit area.

Supplementary Table 5 | Annual water supply volume (×10^8 m^3) of main provinces involved in Zone I.

Year	Heilongjiang		Liaoning		Jilin	
	Total	Groundwater	Total	Groundwater	Total	Groundwater
2004	259.44	103.95	99.19	38.96	130.23	65.00
2005	271.51	113.08	98.39	36.84	133.26	64.27
2006	286.21	114.37	102.90	38.03	141.24	67.01
2007	291.37	124.63	100.78	38.75	142.87	67.17
2008	297.01	127.38	104.08	40.61	142.78	66.58
2009	316.25	136.04	111.09	42.51	142.79	67.35
2010	325.00	146.14	120.04	44.17	143.67	67.58
2011	352.40	149.90	131.20	43.70	144.50	64.30
2012	358.90	161.50	129.80	43.30	142.20	61.30
2013	362.30	167.40	131.50	44.00	142.10	60.00
2014	364.10	167.60	133.00	44.90	141.80	58.40
2015	355.30	157.70	133.60	44.00	140.80	58.60
2016	352.60	166.80	132.50	44.90	135.40	57.00
2017	353.10	163.10	126.70	44.70	131.10	54.50

Data source: China’s Statistical Yearbook on Environment (National Bureau of Statistics).

Supplementary Table 6 | Annual water supply volume (×10^8 m^3) of main provinces involved in Zone II.

Year	Beijing		Tianjin		Hebei	
	Total	Groundwater	Total	Groundwater	Total	Groundwater
2004	34.55	26.80	22.06	7.07	195.87	157.75
2005	34.50	24.90	23.09	6.98	201.78	162.78
2006	34.30	24.34	22.96	6.76	204.00	164.64
2007	34.81	24.19	23.37	6.81	202.50	163.08
2008	35.08	22.94	22.33	6.25	195.02	156.17
2009	35.50	21.80	23.37	6.01	193.72	154.64
2010	35.20	21.19	22.49	5.87	193.68	155.98
2011	36.00	20.90	23.10	5.80	196.00	154.90
2012	35.90	20.40	23.10	5.50	195.30	151.30
2013	36.40	20.00	23.80	5.70	191.30	144.60
2014	37.50	19.60	24.10	5.30	192.80	142.10
2015	38.20	18.20	25.70	4.90	187.20	133.60
2016	38.80	17.50	27.2	4.7	182.6	125.0
2017	39.50	16.60	27.5	4.6	181.6	116.0

Data source: China's Statistical Yearbook on Environment (National Bureau of Statistics).

Supplementary Table 7 | Annual groundwater resource volumes (×10^8 m^3) of main provinces involved in Zone I and Zone II.

Year	Zone I			Zone II		
	Heilongjiang	Liaoning	Jilin	Beijing	Tianjin	Hebei
2004	273.70	94.40	109.60	16.50	5.20	131.10
2005	288.80	122.50	130.20	18.50	4.50	109.70
2006	279.20	104.60	91.60	18.20	4.40	94.30
2007	232.80	86.30	93.40	18.80	4.80	107.20
2008	247.80	99.60	105.40	24.90	5.90	136.30
2009	313.40	97.30	87.60	17.80	5.60	122.70
2010	277.90	141.90	146.80	18.90	4.50	112.90
2011	237.20	112.90	111.90	21.20	5.20	126.20
2012	289.80	147.00	147.40	26.50	7.60	164.80
2013	381.50	160.20	139.40	18.70	5.00	138.80
2014	295.40	120.20	82.30	16.00	3.70	89.30
2015	283.00	127.40	83.20	20.60	4.90	113.60
2016	285.90	154.70	120.90	24.20	6.10	133.70
2017	273.20	133.30	86.60	20.40	5.50	116.30

Data source: China Statistical Yearbook on Environment (National Bureau of Statistics).

Supplementary Table 8 | Proportions of iodine species in different groundwater types.

Well type	Groundwater type	Proportion of IO_3^- and organo-iodine		Proportion of I^-	
		Median	Mean	Median	Mean
Newly constructed	Phreatic	52.61%	50.77%	47.39%	49.23%
	Confined	44.24%	47.11%	55.76%	52.89%
Reconstructed	Phreatic	70.08%	59.39%	29.92%	40.61%
	Confined	65.51%	56.80%	34.49%	43.20%

Supplementary Table 9 | Mean and 95th percentile values of hazard quotient (HQ) via drinking water consumption.

Geo-environmental zones	Iodine deficiency			Iodine excess		
	Mean	95th percentile	Proportion of risk	Mean	95th percentile	Proportion of risk
Zone I	1.23	1.84	84.96%	0.66	0.98	4.42%
Zone II	10.76	47.50	28.10%	5.74	25.33	62.41%
Zone III	5.67	23.31	31.37%	3.02	12.43	47.06%
Zone IV	2.80	7.58	53.91%	1.49	4.04	21.74%
Zone V	0.88	2.03	86.49%	0.47	1.08	5.41%
Zone VI	5.41	13.31	72.22%	2.88	7.10	19.44%
Zone VII	0.58	1.11	89.36%	0.31	0.59	2.13%

Supplementary Table 10 | Iodine state and thyroid nodules prevalence in 36 cities from 31 provinces of China.

Province	Sampled city	Median groundwater iodine content (μg/L)	Median groundwater iodate content (μg/L)	Median groundwater iodide content (μg/L)	Median urine iodine content (μg/L)	Prevalence of thyroid nodules	Prevalence of Subclinical hypothyroidism
Anhui	Hefei	29.52	3.93	22.96	208.64	9.69%	2.49%
Beijing	Beijing	10.97	3.05	4.71	151.52	22.08%	2.85%
Chongqing	Chongqing	9.77	0.83	4.00	193.48	22.37%	2.37%
Fujian	Fuzhou	73.66	9.76	12.52	136.60	30.75%	1.29%
Gansu	Longnan	12.09	2.36	9.21	226.53	17.58%	6.33%
Guangdong	Guangzhou	11.16	3.35	4.99	128.80	41.66%	1.42%
Guangxi	Chongzuo	1.91	0.71	1.01	153.41	13.53%	1.92%
Guizhou	Guiyang	0.85	0.32	0.51	200.30	7.43%	5.50%
Hainan	Haikou and Zhanzhou	13.39	1.45	9.68	151.96	10.80%	1.38%
Hebei	Shijiazhuang and Cangzhou	59.30	7.48	42.07	200.90	17.40%	2.57%
Heilongjiang	Harbin	0.98	0.25	0.37	155.10	22.79%	1.67%

Supplementary Table 10 | Iodine state and thyroid nodules prevalence in 36 cities from 31 provinces of China (*continued*).

Province	Sampled city	Median groundwater iodine content (μg/L)	Median groundwater iodate content (μg/L)	Median groundwater iodide content (μg/L)	Median urine iodine content (μg/L)	Prevalence of thyroid nodules	Prevalence of Subclinical hypothyroidism
Henan	Xinxiang and Kaifeng	245.6	34.16	192.16	225.41	13.61%	4.57%
Hubei	Shiyan	97.16	18.04	60.01	191.84	9.87%	4.00%
Hunan	Changde	30.57	2.75	19.44	172.40	14.63%	3.87%
Inner Mongolia	Hohhot	9.22	3.98	3.81	207.00	24.41%	3.99%
Jiangsu	Xuzhou	246.65	67.94	161.01	304.80	19.06%	3.17%
Jiangxi	Nanchang and Jingdezhen	27.45	8.68	9.37	176.60	9.97%	2.05%
Jilin	Changchun	2.34	0.76	1.15	178.15	20.84%	1.68%
Liaoning	Dalian	0.97	0.33	0.54	149.80	23.73%	2.03%
Ningxia	Guyuan	175.19	58.81	86.10	250.32	30.17%	5.10%
Qinghai	Xining and Haidong	3.92	1.13	1.35	218.42	7.69%	10.43%
Shaanxi	Xi’an	25.85	15.41	7.81	220.59	11.33%	4.31%

Supplementary Table 10 | Iodine state and thyroid nodules prevalence in 36 cities from 31 provinces of China (*continued*).

Province	Sampled city	Median groundwater iodine content (μg/L)	Median groundwater iodate content (μg/L)	Median groundwater iodide content (μg/L)	Median urine iodine content (μg/L)	Prevalence of thyroid nodules	Prevalence of Subclinical hypothyroidism
Shandong	Taian	5.47	2.95	0.99	137.90	10.79%	2.23%
Shanghai	Shanghai	184.55	49.55	83.77	164.15	24.78%	2.29%
Shanxi	Taiyuan	4.83	2.64	0.82	211.85	11.70%	2.34%
Sichuan	Chengdu	2.86	0.87	1.36	172.10	10.87%	3.59%
Tianjin	Tianjin	40.60	10.69	18.67	142.20	30.12%	6.69%
Tibet	Lhasa	1.739	0.36	0.58	171.40	25.48%	1.71%
Xinjiang	Turpan	3.302	2.06	1.07	147.98	33.10%	3.29%
Yunnan	Kunming	2.098	0.54	1.08	177.70	21.47%	4.54%
Zhejiang	Hangzhou	13.4	4.35	1.84	216.93	15.29%	3.02%

Supplementary Table 11 | Proportion of monitored wells at iodine-induced risk at provincial level in China.

Province	Monitored wells with iodine-induced risks		Province	Monitored wells with iodine-induced risks	
	Deficiency	Excess		Deficiency	Excess
Hebei	35.80%	48.15%	Ningxia	20.00%	60.00%
Henan	34.69%	61.22%	Hainan	25.00%	25.00%
Inner Mongolia	14.29%	42.86%	Hunan	0.00%	80.00%
Heilongjiang	88.89%	0.00%	Sichuan	100.00%	0.00%
Beijing	44.44%	38.89%	Guangxi	40.00%	30.00%
Shanxi	55.56%	22.22%	Jiangxi	40.00%	40.00%
Liaoning	97.50%	0.00%	Guangdong	12.50%	50.00%
Shandong	45.07%	45.07%	Hubei	25.00%	75.00%
Shaanxi	65.38%	23.08%	Fujian	12.50%	62.50%
Jilin	86.05%	4.65%	Guizhou	83.33%	16.67%
Gansu	70.59%	5.88%	Yunnan	100.00%	0.00%

Supplementary Table 11 | Proportion of monitored wells at iodine-induced risk at provincial level in China (*continued*).

Province	Monitored wells with iodine-induced risks		Province	Monitored wells with iodine-induced risks	
	Deficiency	Excess		Deficiency	Excess
Xinjiang	69.57%	21.74	Chongqing	40.00%	20.00%
Qinghai	85.00%	0.00%	Jiangsu	0.00%	92.86%
Tianjin	20.00%	66.67%	Zhejiang	40.00%	40.00%
Anhui	14.29%	71.43%	Shanghai	0.00%	100.00%
Tibet	95.83%	4.17%			

Supplementary Table 12 | Median groundwater iodine content at provincial level in China.

Province	Median content (μg/L)	Province	Median content (μg/L)
Hebei	13.16	Ningxia	20.59
Henan	33.42	Hainan	13.52
Inner Mongolia	14.74	Hunan	30.57
Heilongjiang	1.89	Sichuan	2.86
Beijing	10.97	Guangxi	13.98
Shanxi	7.18	Jiangxi	12.94
Liaoning	0.86	Guangdong	32.29
Shandong	10.34	Hubei	97.16
Shaanxi	4.93	Fujian	21.40
Jilin	2.34	Guizhou	2.18
Gansu	3.41	Yunnan	3.20
Xinjiang	4.58	Chongqing	9.77
Qinghai	4.59	Jiangsu	78.75
Tianjin	40.60	Zhejiang	13.40
Anhui	26.84	Shanghai	184.55
Tibet	2.81		

Supplementary Table 13 | Average proportions of groundwater used for water supply over 5 years (2010 ~ 2014 and 2015 ~ 2019) at provincial level in China.

Province	Proportion of groundwater		Province	Proportion of groundwater	
	2010 ~2014	2015 ~2019		2010 ~2014	2015 ~2019
Hebei	77.27%	62.97%	Ningxia	7.72%	8.54%
Henan	57.94%	50.59%	Hainan	7.20%	6.46%
Inner Mongolia	49.53%	45.98%	Hunan	5.63%	4.48%
Heilongjiang	44.95%	45.19%	Sichuan	7.34%	4.37%
Beijing	56.46%	42.56%	Guangxi	3.69%	3.66%
Shanxi	51.41%	41.47%	Jiangxi	3.80%	3.28%
Liaoning	43.61%	41.33%	Guangdong	3.91%	3.16%
Shandong	40.23%	37.46%	Hubei	3.21%	2.73%
Shaanxi	37.96%	35.15%	Fujian	2.87%	2.71%
Jilin	34.14%	34.30%	Guizhou	2.89%	2.57%
Gansu	21.71%	21.92%	Yunnan	3.43%	2.43%
Xinjiang	19.47%	19.62%	Chongqing	1.96%	1.55%
Qinghai	14.67%	18.49%	Jiangsu	1.68%	1.38%
Tianjin	24.19%	16.44%	Zhejiang	1.66%	0.68%
Anhui	10.92%	10.76%	Shanghai	0.10%	0.00%
Tibet	10.53%	10.57%			

Data source: China's Statistical Yearbook on Environment (National Bureau of Statistics).

Supplementary Table 14 | Iodine content in drinking water of China.

Province	Number of sampled counties	Median content (µg/L)	Number of counties			
			<10 µg/L	10~100 µg/L	100~300 µg/L	>300 µg/L
Beijing	16	3.4	16	0	0	0
Tianjin	16	5.1	12	3	1	0
Hebei	172	4.7	120	31	17	4
Shanxi	119	5.0	88	30	1	0
Inner Mongolia	104	6.7	73	31	0	0
Liaoning	105	3.7	95	10	0	0
Jilin	64	4.6	57	7	0	0
Heilongjiang	132	5.0	120	12	0	0
Shanghai	16	2.7	15	1	0	0
Jiangsu	103	7.3	79	22	1	1
Zhejiang	89	2.1	83	6	0	0
Anhui	105	4.3	77	18	10	0
Fujian	88	2.2	83	5	0	0
Jiangxi	100	2.5	100	0	0	0
Shandong	137	9.2	74	49	11	3
Henan	170	9.6	94	65	11	0

Data source: Investigation on iodine content in drinking water of China (National Health Commission of the People's Republic of China).

Supplementary Table 14 | Iodine content in drinking water of China (*continued*).

Province	Number of sampled counties	Median content (μg/L)	Number of counties			
			<10 μg/L	10~100 μg/L	100~300 μg/L	>300 μg/L
Hubei	103	3.7	101	2	0	0
Hunan	132	2.5	121	10	1	0
Guangdong	128	5.1	107	21	0	0
Guangxi	112	3.3	107	5	0	0
Hainan	25	6.0	25	0	0	0
Chongqing	39	1.5	39	0	0	0
Sichuan	189	2.1	188	1	0	0
Guizhou	91	1.8	91	0	0	0
Yunnan	129	1.3	129	0	0	0
Tibet	74	2.2	74	0	0	0
Shaanxi	110	4.6	77	32	0	0
Gansu	89	2.2	89	0	0	0
Qinghai	43	1.7	42	1	0	0
Ningxia	23	6.4	18	5	0	0
Xinjiang	113	3.8	104	9	0	0

Data source: Investigation on iodine content in drinking water of China (National Health Commission of the People’s Republic of China).

Supplementary Table 15 | Proportion of groundwater iodine species at provincial level in China.

Province	Proportion of iodine species			Province	Proportion of iodine species		
	I^-	IO_3^-	organo-iodine		I^-	IO_3^-	organo-iodine
Hebei	60.52%	13.99%	25.50%	Ningxia	37.05%	38.31%	24.64%
Henan	65.28%	17.67%	17.05%	Hainan	57.88%	16.67%	25.45%
Inner Mongolia	56.21%	23.29%	20.50%	Hunan	53.26%	19.54%	27.21%
Heilongjiang	44.98%	28.59%	26.43%	Sichuan	47.54%	30.50%	21.96%
Beijing	42.97%	27.84%	29.19%	Guangxi	50.75%	25.85%	23.41%
Shanxi	22.76%	57.96%	19.28%	Jiangxi	24.50%	36.44%	39.06%
Liaoning	60.69%	29.10%	10.21%	Guangdong	55.37%	24.49%	20.15%
Shandong	45.39%	26.85%	27.75%	Hubei	61.76%	18.56%	19.68%
Shaanxi	37.55%	47.98%	14.47%	Fujian	33.34%	40.31%	26.35%
Jilin	49.18%	32.44%	18.37%	Guizhou	47.01%	29.49%	23.50%
Gansu	36.34%	42.01%	21.66%	Yunnan	33.66%	26.60%	39.74%

Supplementary Table 15 | Proportion of groundwater iodine species at provincial level in China (*continued*).

Province	Proportion of iodine species			Province	Proportion of iodine species		
	I^-	IO_3^-	organo-iodine		I^-	IO_3^-	organo-iodine
Xinjiang	36.55%	48.28%	15.17%	Chongqing	53.64%	14.27%	32.09%
Qinghai	37.02%	33.84%	29.15%	Jiangsu	70.65%	16.46%	12.89%
Tianjin	45.98%	26.33%	27.70%	Zhejiang	13.70%	32.49%	53.81%
Anhui	71.20%	18.59%	10.22%	Shanghai	80.95%	10.30%	8.74%
Tibet	35.46%	27.74%	36.80%				

Supplementary Table 16 | Key parameters for iodine-induced non-carcinogenic risk assessment.

Parameter	Units	Value	Parameter	Units	Value
Concentration of iodine (C_w)	μg/L	Present study	Body weight (BW)	kg	60
Ingestion rate (IR_w)	L/day	2	Average time (AT)	day	10950
Exposure frequency (EF)	day/year	365	Reference dose of iodine (RfD) [a]	mg/(kg·day)	Lower limit: 1.3<br>Upper limit: 2.5
Exposure duration (ED)	year	30			

[a] Reference dose of iodine is based on recommended iodine intake specified by the World Health Organization.

Supplementary Table 17 | Salt iodization levels in different provinces of China[8].

Province	Average iodine content (mg/kg)	Iodized salt coverage	Province	Average iodine content (mg/kg)	Iodized salt coverage
Hebei	25.0	93.87%	Ningxia	30.0	98.05%
Henan	30.0	98.26%	Hainan	25.0	97.77%
Inner Mongolia	25.0	96.17%	Hunan	30.0	97.35%
Heilongjiang	30.0	97.92%	Sichuan	30.0	97.21%
Beijing	25.0	95.40%	Guangxi	25.0	99.25%
Shanxi	25.0	96.19%	Jiangxi	25.0	99.96%
Liaoning	27.5	96.54%	Guangdong	25.0	98.14%
Shandong	25.0	96.26%	Hubei	25.0	95.97%
Shaanxi	25.0	98.27%	Fujian	25.0	90.35%
Jilin	25.0	97.66%	Guizhou	30.0	99.62%
Gansu	30.0	99.78%	Yunnan	25.0	99.66%
Xinjiang	30.0	99.77%	Chongqing	30.0	99.08%
Qinghai	30.0	99.66%	Jiangsu	25.0	99.13%
Tianjin	30.0	79.01%	Zhejiang	25.0	64.24%
Anhui	25.0	99.46%	Shanghai	30.0	77.82%
Tibet	30.0	97.53%			

Supplementary Table 18 | Dietary reference values for iodine.

Dietary reference values	Value (μg/day)
Recommended nutrient intake of iodine [a]	150
Tolerable upper iodine intake level [b]	600

[a] Recommended nutrient intake of iodine is provided by the World Health Organization.

[b] Tolerable upper iodine intake level is established by the Institute of Medicine.

Supplementary Table 19 | Recommended limits for identifying high iodine groundwater.

Recommended limits (RL)	Value (μg/L)
RL_R [a]	75
RL_U [b]	300

[a] RL_R is calculated based on the recommended nutrient intake of iodine (RNI).

[b] RL_U is calculated based on the tolerable upper iodine intake level (UL).

Supplementary References

1. Fuge, R. & Johnson, C. C. Iodine and human health, the role of environmental geochemistry and diet, a review. *Appl. Geochem.* **63**, 282-302 (2015).
2. Li, J. X. et al. Fluoride and iodine enrichment in groundwater of North China Plain: Evidences from speciation analysis and geochemical modeling. *Sci. Total Environ.* **598**, 239-248 (2017).
3. Xue, X. B. et al. Impacts of sediment compaction on iodine enrichment in deep aquifers of the North China Plain. *Water Res.* **159**, 480-489 (2019).
4. Xue, X. B. et al. Effects of depositional environment and organic matter degradation on the enrichment and mobilization of iodine in the groundwater of the North China Plain. *Sci. Total Environ.* **686**, 50-62 (2019).
5. Hou, X. L. et al. A review on speciation of iodine-129 in the environmental and biological samples. *Anal. Chim. Acta* **632**, 181-196 (2009).
6. Li, Y. Z. et al. Efficacy and Safety of Long-Term Universal Salt Iodization on Thyroid Disorders: Epidemiological Evidence from 31 Provinces of Mainland China. *Thyroid* **30**, 568-579 (2020).
7. National Health Commission of China *Investigation on iodine content in drinking water of China.*

http://www.nhc.gov.cn/jkj/s5874/201905/bb1da1f5e47040e8820b9378e6db4bd3.shtml (2019).

8. Chinese Medical Association (Local Epidemiology Branch), Chinese Nutrition Society & Chinese Society of Endocrinology. *The Iodine Supplementation Guidelines for Chinese Residents*. http://www.nhc.gov.cn/ewebeditor/uploadfile/2018/05/20180515144010634.pdf (2018).
