# Supplementary material for: Deficiency and excess of groundwater iodine and their health associations
Source: Nat Commun. 2022 Nov 29;13:7354. doi: 10.1038/s41467-022-35042-6 (PMC9708681; doi:10.1038/s41467-022-35042-6)
Supplement: Supplementary file 2 — Reporting Summary [file 41467_2022_35042_MOESM2_ESM.pdf]

nature portfolio

Corresponding author(s): Jinren Ni

Last updated by author(s): Nov 16, 2022

Reporting Summary

Nature Portfolio wishes to improve the reproducibility of the work that we publish. This form provides structure for consistency and transparency in reporting. For further information on Nature Portfolio policies, see our Editorial Policies and the Editorial Policy Checklist.

Statistics

For all statistical analyses, confirm that the following items are present in the figure legend, table legend, main text, or Methods section.

n/a	Confirmed	
[ ]	[x]	The exact sample size (n) for each experimental group/condition, given as a discrete number and unit of measurement
[ ]	[x]	A statement on whether measurements were taken from distinct samples or whether the same sample was measured repeatedly
[ ]	[x]	The statistical test(s) used AND whether they are one- or two-sided<br>*Only common tests should be described solely by name; describe more complex techniques in the Methods section.*
[x]	[ ]	A description of all covariates tested
[ ]	[x]	A description of any assumptions or corrections, such as tests of normality and adjustment for multiple comparisons
[ ]	[x]	A full description of the statistical parameters including central tendency (e.g. means) or other basic estimates (e.g. regression coefficient) AND variation (e.g. standard deviation) or associated estimates of uncertainty (e.g. confidence intervals)
[ ]	[x]	For null hypothesis testing, the test statistic (e.g. F, t, r) with confidence intervals, effect sizes, degrees of freedom and P value noted<br>*Give P values as exact values whenever suitable.*
[x]	[ ]	For Bayesian analysis, information on the choice of priors and Markov chain Monte Carlo settings
[x]	[ ]	For hierarchical and complex designs, identification of the appropriate level for tests and full reporting of outcomes
[x]	[ ]	Estimates of effect sizes (e.g. Cohen's d, Pearson's r), indicating how they were calculated

Our web collection on statistics for biologists contains articles on many of the points above.

Software and code

Policy information about availability of computer code

Data collection	No software was used.
Data analysis	Origin 2018, IBM SPSS Statistics 20.0, GeoDa 1.14 and ArcGIS Map 10.4 were used to analyse the data in this study.

For manuscripts utilizing custom algorithms or software that are central to the research but not yet described in published literature, software must be made available to editors and reviewers. We strongly encourage code deposition in a community repository (e.g. GitHub). See the Nature Portfolio guidelines for submitting code & software for further information.

Data

Policy information about availability of data

All manuscripts must include a data availability statement. This statement should provide the following information, where applicable:

- Accession codes, unique identifiers, or web links for publicly available datasets
- A description of any restrictions on data availability
- For clinical datasets or third party data, please ensure that the statement adheres to our policy

All data supporting the findings of this study, including China’s national distribution maps of iodine and its species, iodine-induced health risk, and epidemiological data on thyroid diseases, are available within the paper and its supplementary information file. The geographical information of sampling sites and concentration of groundwater iodine have been deposited in the figshare database [https://doi.org/10.6084/m9.figshare.21507528.v1]. The map data used in this study is available in the GeoCloud Database developed by China Geological Survey [https://geocloud.cgs.gov.cn/].

Human research participants

Policy information about studies involving human research participants and Sex and Gender in Research.

Reporting on sex and gender	This study does not involve human research participants, vertebrate animals or cell lines.
Population characteristics	This study does not involve human research participants.
Recruitment	This study does not involve human research participants.
Ethics oversight	This study does not involve human research participants, vertebrate animals or cell lines.

Note that full information on the approval of the study protocol must also be provided in the manuscript.

Field-specific reporting

Please select the one below that is the best fit for your research. If you are not sure, read the appropriate sections before making your selection.

☐ Life sciences ☐ Behavioural & social sciences ☒ Ecological, evolutionary & environmental sciences

For a reference copy of the document with all sections, see nature.com/documents/nr-reporting-summary-flat.pdf

Ecological, evolutionary & environmental sciences study design

All studies must disclose on these points even when the disclosure is negative.

Study description	This study stressed the urgency of accurate iodine supply depending on spatial heterogeneity, and highlighted the importance of integrated iodine provision to mitigating risks in iodine-deficient and -excess areas globally.
Research sample	A comprehensive dataset derived from 686 standard monitoring wells distributed in 31 provinces of China. The sampling sites covered China's seven groundwater geo-environmental zones, which could comprehensively reflect the spatial distribution of groundwater iodine.<br>The surveyed data on population iodine nutritional level and thyroid diseases were obtained based on a national cross-sectional study, covering 78490 enrolled participants (aged 18 or older) from 36 sampled cities in 31 provinces of China during 2015 ~ 2017.
Sampling strategy	Based on China's seven groundwater geo-environmental zones, 686 groundwater samples from standard monitoring wells located in 31 provinces of China. The sampling sites covered almost the whole of China including typical terrain (plain, basin, and plateau), major river basins (e.g., Yangtze, Yellow, Huai, Hai, and Pearl), and dominant urban areas (Beijing, Shanghai, Guangzhou, etc.). The layout of sampling sites was in accordance with Technical Specifications for Environmental Monitoring of Groundwater (HJ/T 164-2004). The monitoring campaign also took account of drinking water sources, human interference (e.g., pollution and agricultural irrigation), and seawater intrusion.<br>Therefore, the 686 groundwater samples selected in this study are representative, which could help to reflect natural and anthropogenic effects on spatial distribution of groundwater iodine and its species in China.
Data collection	All groundwater sampling followed China's standard procedure for the environmental monitoring of groundwater (HJ 494-2009). Groundwater samples were collected by trained members of the research group.<br>Before sample collection, each monitoring well was purged by pumping out groundwater with an outflow discharge below 100 mL/min. Outflow water quality indexes (pH, temperature, electrical conductivity, oxidation-reduction potential, dissolved oxygen, and turbidity) were measured using a portable water quality meter (WTW Multi 3630 IDS) every 5 ~ 15 minutes until the indexes became stable (≤ ± 10%) for three consecutive measurements. Groundwater samples were then collected, filtered through a 0.45 µm membrane, and stored timely in 250 mL iodine-free HDPE containers covered with aluminum foil to avoid light. During transportation and laboratory storage, samples were kept frozen in a refrigerator.<br>Alkalinity was analyzed by titration with 0.025 M HCl within 24 h after sampling. Anion contents, including chloride (Cl-) and sulfate radical (SO42-), were analyzed using an ion chromatography system. Cations, such as potassium (K+), calcium (Ca2+), sodium (Na+), and magnesium (Mg2+), were determined using an inductively coupled plasma-optical emission spectrometer.<br>Total iodine (TI = TII + Organo-iodine) concentration was determined by inductively coupled plasma-mass spectrometry. Total inorganic iodine (TII = I- + IO3-) content in groundwater was determined by gas chromatography-mass spectrometry with derivatization. Organo-iodine content was calculated from the difference between TI and TII. Experiments were conducted by trained members of the research group.
Timing and spatial scale	Our groundwater iodine database includes data from 686 groundwater samples, which were collected continuously from standard monitoring wells located in 31 provinces of China during 2016 to 2017.<br>Sampling sites were based on China's seven groundwater geo-environmental zones: Northeast Plain-Mountain Zone, Huanghuaihai-Yangtze River Delta Plain Zone, South China Bedrock Foothill Zone, Northwest Loess Plateau Zone, Southwest China Karst Rock Mountain Zone, Northwest Arid Desert Zone, and Qinghai-Tibet Plateau Alpine Frozen Soil Zone. The sample collection and experimental analysis were conducted simultaneously.
Data exclusions	To control data quality, we removed outliers in the database. First, we detected total inorganic iodine and total iodine by gas

chromatography-mass spectrometry and inductively coupled plasma-mass spectrometry respectively. Samples with the total inorganic iodine content greater than total iodine content were considered outliers. We removed 23 outliers, which accounts for 3.2% of the total data points.

Reproducibility	Parallel experiments were designed for 20% groundwater samples to ensure the accuracy of experimental results, and the relative deviation in parallel samples was invariably below 20%. All attempts to repeat the experiment were successful.
Randomization	We detected total iodine and its species contents of all collected groundwater samples.<br>According to the current National Standards in China for water-borne iodine-deficiency/excess endemic areas, iodine content in groundwater could be divide into the following four categories: low, TI < 10 μg/L; medium, 10 ≥ TI > 100 μg/L; high, 100 ≥ TI > 300 μg/L; and very high, TI > 300 μg/L.
Blinding	There was no experimenter involved in this study.

Did the study involve field work? ☐ Yes ☒ No

Reporting for specific materials, systems and methods

We require information from authors about some types of materials, experimental systems and methods used in many studies. Here, indicate whether each material, system or method listed is relevant to your study. If you are not sure if a list item applies to your research, read the appropriate section before selecting a response.

Materials & experimental systems

n/a	Involved in the study
☒	☐ Antibodies
☒	☐ Eukaryotic cell lines
☒	☐ Palaeontology and archaeology
☒	☐ Animals and other organisms
☒	☐ Clinical data
☒	☐ Dual use research of concern

Methods

n/a	Involved in the study
☒	☐ ChIP-seq
☒	☐ Flow cytometry
☒	☐ MRI-based neuroimaging
